# The impact of US–China tensions on US science: Evidence from the NIH investigations

**DOI:** 10.1073/pnas.2301436121

**Published:** 2024-04-30

**Authors:** Ruixue Jia, Margaret E. Roberts, Ye Wang, Eddie Yang

**Affiliations:** ^a^School of Global Policy and Strategy, University of California San Diego, La Jolla, CA 92093; ^b^Department of Political Science, University of California San Diego, La Jolla, CA 92093; ^c^Halıcıoğlu Data Science Institute, University of California San Diego, La Jolla, CA 92093; ^d^Department of Political Science, University of North Carolina, Chapel Hill, NC 17599

**Keywords:** international relations, science, innovation

## Abstract

Amid the discourse on foreign influence investigations in research, this study examines the impact of NIH-initiated investigations starting in 2018 on U.S. scientists’ productivity, focusing on those collaborating with Chinese peers. Using publication data from 2010 to 2021, we analyze over 113,000 scientists and find that investigations coincide with reduced productivity for those with China collaborations compared to those with other international collaborators, especially when accounting for publication impact. The decline is particularly pronounced in fields that received greater preinvestigation NIH funding and engaged more in U.S.–China collaborations. Indications of scientist migration and broader scientific progress implications also emerge. We also offer insights into the underlying mechanisms via qualitative interviews.

## Significance

While there has been much discussion about recent U.S. investigations of foreign influence in scientific research, very little work has quantified how these investigations have affected the productivity of U.S. scientists. We focus on NIH investigations initiated in 2018 and offer quantitative evidence, drawing from comprehensive PubMed and Dimensions data (2010–2021), revealing detrimental effects. In tandem with quantitative findings, insights derived from qualitative interviews shed light on the multifaceted impact experienced by scientists. Our findings highlight that scientific production and progress can be very sensitive to political dynamics, echoing themes explored in economics, science and technology studies, and political science.

Science is becoming more collaborative, and scientific collaboration is increasingly international (1–4). From 2008 to 2018, the percentage of science and engineering papers with authors from institutions in different countries has increased from 17 to 23% (5). International collaborations in science have resulted in great achievements, exemplified by the International Space Station and the completion of the Human Genome Project. A large literature has documented how international collaboration and talent flows can facilitate progress in science (6–11).

However, science is never isolated from politics, and is often affected by national and international policies (12–18). In recent years, due to political tensions between the U.S. and China, scientific collaborations between U.S. and Chinese academic institutions have come under increasing scrutiny by U.S. policymakers. The U.S. Department of Justice started the China Initiative, which ran from 2018–2022, aimed at countering national security threats from China, with a particular focus on intellectual property and technology.^*^ Also in 2018, the NIH began contacting institutions of higher education about investigations of hundreds of scientists, largely for failure to disclose receipt of foreign resources on federal research grants.^†^ While the investigations were not specific to China, the vast majority of investigated cases involved receipt of resources from China. As of July 2021, according to disclosed cases, these investigations involved at least 93 institutions of higher education and 214 scientists, 90% of which involved receipt of resources or activities in China.^‡^ Some cases resulted in suspension of funding, termination of employment, and in rare cases criminal investigations of scientists.^§^

While the merits of the China Initiative and NIH investigations have been widely discussed (19–22), much less is known about the impact of these policies on U.S. production of science. In this paper, we study the impact of the NIH investigations on U.S. production of science by examining the publications of U.S. scientists in the fields of life sciences. Because the focus of the scrutiny has been on researchers with academic collaborations in China, we closely examine scientists with a history of collaborating with institutions in China. Using large-scale publication databases, we investigate whether life scientists at U.S. institutions with a history of collaborating with scientists in China have been less productive since the onset of the NIH investigations, relative to their colleagues in the United States who have a history of collaborating with scientists from other countries.^¶^

**Fig. 1.**
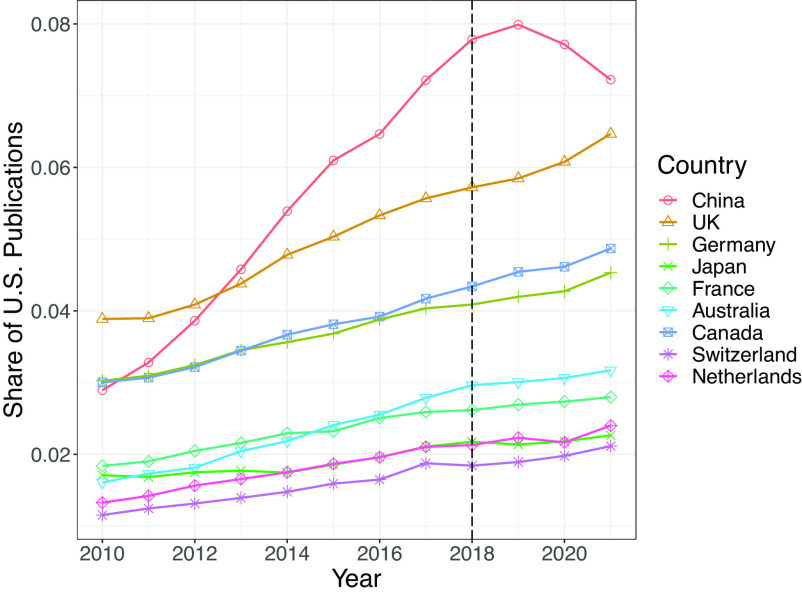
Collaboration as Share of Total U.S. PubMed Publications *Note:* The data are based on publications indexed by PubMed from Dimensions. Each line represents U.S. collaboration with a given country in PubMed publications as its share of total U.S. PubMed publications. Note that the data include all scientists in the Dimensions database, not just those included in the data we describe below.

We focus on life sciences for both conceptual and empirical reasons. Conceptually, the NIH’s focus is on funding scientists in life sciences. While other federal research agencies also conducted investigations about foreign influence in research, the NIH was the first and to our knowledge most frequent federal agency to conduct them. Empirically, there exist multiple data sources on publications in these fields, making quantitative analysis of publication trends tractable. Specifically, we employ two data sources: the PubMed database (https://pubmed.ncbi.nlm.nih.gov/) that covers publications on life sciences and biomedical topics and is maintained by institutions located at the NIH and the Dimensions database (https://www.dimensions.ai/) that covers publications from all scientific fields. As shown in Fig. 1, China has been the most important collaborator of the United States in life sciences since 2013. However, compared with U.S. collaborations with other countries, U.S.–China collaborations appear to slow down in 2019, which coincides with the NIH investigations, and have turned downward since then. We observe a similar pattern when examining publications by Chinese scientists, suggesting that U.S.–China tensions can affect both countries (*SI Appendix*, Fig. S1).

To estimate the causal effect of the investigations on scientists with previous collaborations with institutions in China, we employ a difference-in-differences approach. Specifically, we define the treated and control groups of Principal Investigators (PI) based on the publication records during 2010–2014.^#^ We assume that those who had collaborations with scholars in China during this period are “treated,” in that they are particularly affected by the investigations, and use those who collaborated with scholars from other non-U.S. countries as the control group. In our data, 35,140 PIs belong to the treated group and 78,086 PIs to the control group. Then, using publication data during 2015–2021, we examine how the quantity and citations of publications differ between treated and control groups before and after the NIH investigations in 2018. To consider possible differences in individual characteristics and career paths, our analyses control for individual fixed effects, year fixed effects, and consider year-specific impacts of ethnicity and pretreatment productivity. We complement our analyses with reweighting and matching strategies in which we ensure the covariates are comparable between the treated and control groups.

We find that the PubMed publications of scientists with a history of collaborating with scientists in China experienced a decline after 2018, compared with their counterparts without collaborators in China. While the magnitude of the decline in quantity is small (2.1%), the effect becomes sizable (10.1%) once we consider the impact of publications and employ citations of publications as the outcome. This finding suggests that the treated scientists were affected not only in terms of quantity but also the influence of their research output. For non-PubMed publications, we find a minimal increase in quantity but a sizable decline in citations (5.7%). Together, in terms of total publications, the treated scientists experienced a decline of 10.5% in publication citations.

Our main finding is robust to using alternative measures of productivity (e.g., studying the number of hit papers and considering journal rankings) and examining the intensity of treatment. When examining the pretrends, we find that the productivity of the treated scientists was not on a different trend but declined after the investigations. When looking at different collaborations, we find that it seems difficult to substitute U.S.–China collaborations with other international collaborations, at least in the period we are studying. While considering migration of scientists does not affect our main finding, we document suggestive evidence that the treated PIs are more likely to migrate out of the United States after 2018.

An important challenge for our studied period is the influence of COVID-19 on scientific productivity. Although all scientists in our sample have international collaborations that can be affected by COVID-19, we are still concerned whether COVID-19 policies in China could be a confounding factor. We should note that we observe effects from the treatment even before the pandemic.^‖^ To partially address this challenge, we take a closer look at publications by institutions, scientist characteristics, and research fields. We document three patterns. First, motivated by the discussion on racial profiling in the China Initiative (20), we examine whether Asian scientists are more adversely affected. We find that among the treated, Asian scientists are more affected for both NIH-funded and China-funded publications. Second, the adverse effects appear to apply to most of the institutions and scientists of different productivity and career stages, suggesting that this is a broad phenomenon and is not limited to a narrow group of scientists. Third, to investigate which fields are more affected, we calculate the importance of NIH funding and U.S.–China collaboration by fields and estimate the impact in each field. We find that the fields where NIH funding is more important and had more U.S.–China collaborations experienced a larger decline. These patterns further support that our findings are driven by U.S.–China tensions rather than other shocks (including COVID-19) during this period that are orthogonal to NIH funding or U.S.–China collaboration.^**^

Further, we provide suggestive evidence that our findings are relevant for science at the aggregate level for both the United States and China. Specifically, we correlate the changes in scientific output by field in China and the United States (relative to 48 other countries) with our estimated impact of the NIH investigations by field. We find that the fields that we identify to be more adversely affected by the NIH investigations experienced slower growth in scientific output than fields that are less affected. This association holds both for China and the United States, suggesting that both countries have been negatively affected.

Finally, to shed light on the underlying mechanisms, we complement our quantitative analyses with interviews of scientists. The interviews of 12 scientists suggest that the short-run impacts we document stem from three channels: a direct effect of NIH funding reduction, a decline in access to human capital, including students and collaborators, from China, and a chilling effect on collaborating with institutions in China. Multiple scientists emphasize that they are less willing to start new projects with scientists in China, which has forced them to reorient their work toward other topics, and has been costly in terms of productivity. These channels suggest that our findings above may underestimate the impacts in the long run, since it takes time for the reduction of new joint projects to appear in our data.

The NIH investigations have attracted much attention from scientists and the public. Yet, the consequences of these investigations have been little understood. Recent studies on U.S.–China tensions investigate the return migration of Chinese-origin scientists from the United States back to China and the productivity of Chinese scientists (24, 25). Our study provides a step toward depicting how scientific production is affected. Admittedly, our characterization focuses on the outcomes in the short run and additional impacts are likely to unfold in the long run.

Our research is related to an extensive literature in economics, science and technology studies, and political science that investigates how constraints on information, collaboration, and talent mobility impact scientific progress and innovation. Besides the literature mentioned above, researchers have also characterized the rapid growth of collaborations among scientists located in different countries, especially those between the United States and China (26–28). With political tensions between the United States and China increasing, it is not clear how scientific collaboration between the two countries will evolve. Our study provides evidence that scientific production and collaboration can be very sensitive to political pressure.

## 1. Descriptive Evidence and Research Design

We focus on publications in the biomedical fields and life sciences in the period 2010–2021. We start with publications indexed by PubMed, an online resource from the National Library of Medicine that archives literature in the biomedical and life sciences. To obtain the metadata associated with these publications, we make use of another database, Dimensions, that provides metadata such as author affiliations, citation counts, and fields of study. As each author in the Dimensions database is indexed by a unique author identifier, we are able to track each author’s publication record.

### 1.1. Defining Treatment and Control Groups.

We define the treated group as individuals in our sample of U.S. medical and life scientists who had at least one paper collaborated with some scholar from an institution in China in the period between 2010 and 2014 (See *Materials and Methods* for sample construction details). In our data, 35,140 PIs belong to the treated group. In our analyses, we also consider the intensity of treatment by measuring the number of China-collaborations during the period of 2010–2014.

The control group consists of those who both 1) had at least one paper collaborated with some scholar from a foreign country other than China from 2010 to 2014 and 2) had no collaboration with scholars in China in the pretreatment period from 2010 to 2018. We define the control group in this way to make it more comparable to the treated group because scientists who have international collaborators in our data tend to be more productive than those who do not.^††^ In our data, 78,086 PIs belong to the control group.

We consider 2019 as the first year under treatment. On August 20, 2018, the Director of the NIH, Francis Collins, sent out an open letter to U.S. universities, calling for investigations into foreign influence in research and undisclosed foreign funding. This date marks the beginning of the treatment we are interested in and was also frequently cited in our interviews as the year that scientists began to feel pressure on their collaborations with scientists in China. Nevertheless, there is a time gap between research and publication, meaning that the impacts of the investigations may not be reflected immediately, which is why we select 2019 as the first year under treatment.

### 1.2. Summary Statistics.

We present summary statistics by treated and control groups in Table 1. Our main analyses focus on two measures of productivity: quantity of publications in a given year and total citations for publications in that year,^‡‡^ the latter of which can be considered as a impact-weighted productivity measure because it is the number of publications weighted by citations. In addition, we employ additional metrics such as average citations and the number of hit papers for robustness.

**Table 1.** Summary statistics

	Control group		Treated group	
	Mean	SD	Mean	SD
A. Pretreatment (2015–2018)				
Total citations	114.0	292.5	383.8	970.2
PubMed citations	105.8	287.0	341.9	926.5
NIH citations	51.4	195.8	194.5	721.4
PubMed publications	3.0	4.0	5.9	7.6
B. Posttreatment (2019–2021)				
Total citations	47.9	175.9	147.4	439.4
PubMed citations	44.1	172.2	127.5	408.7
NIH citations	22.4	131.6	71.1	306.2
PubMed publications	3.0	4.8	5.8	8.5
C. △ln(post)−ln(pre)				
Total citations	−0.87		−0.96	
PubMed citations	−0.88		−0.97	
NIH citations	−0.83		−1.01	
PubMed publications	0.00		−0.02	
Asian researchers	9,014		10,015	
No. of obs.	78,086		35,140	

As shown in Panels *A* and *B*, treated scientists are on average more productive than control scientists and are better cited. This partly reflects the prominence of collaborations with China among the more productive U.S. scientists. These data also reveal that citations from publications indexed by PubMed account for the majority of total citations and citations from NIH-supported publications account for about half of the PubMed citations among the scientists in our sample.

Panel *C* presents the change in citations and publications for the treated and control groups. As more recent publications have fewer citations, there exists a general decline in citations of publications over time. However, the decline appears systematically larger for the treated scientists than their control counterparts. For instance, for the treated scientists, the relative decline is 9% more in terms of total citations, 9% more in terms of PubMed citations, and 18% more in terms of NIH citations. The difference in PubMed publications exhibits a similar pattern but the magnitude is smaller.

In addition, using the prediction method in ref. 29, we estimate whether a scientist is of Asian heritage by using his or her family name (see more details about the prediction method in *SI Appendix*, section 2). In our sample, the shares of Asian scientists in the treatment and control groups are 28.5% and 11.5% respectively, reflecting that Asian scientists are more likely to collaborate with scientists in China.

**Fig. 2.**
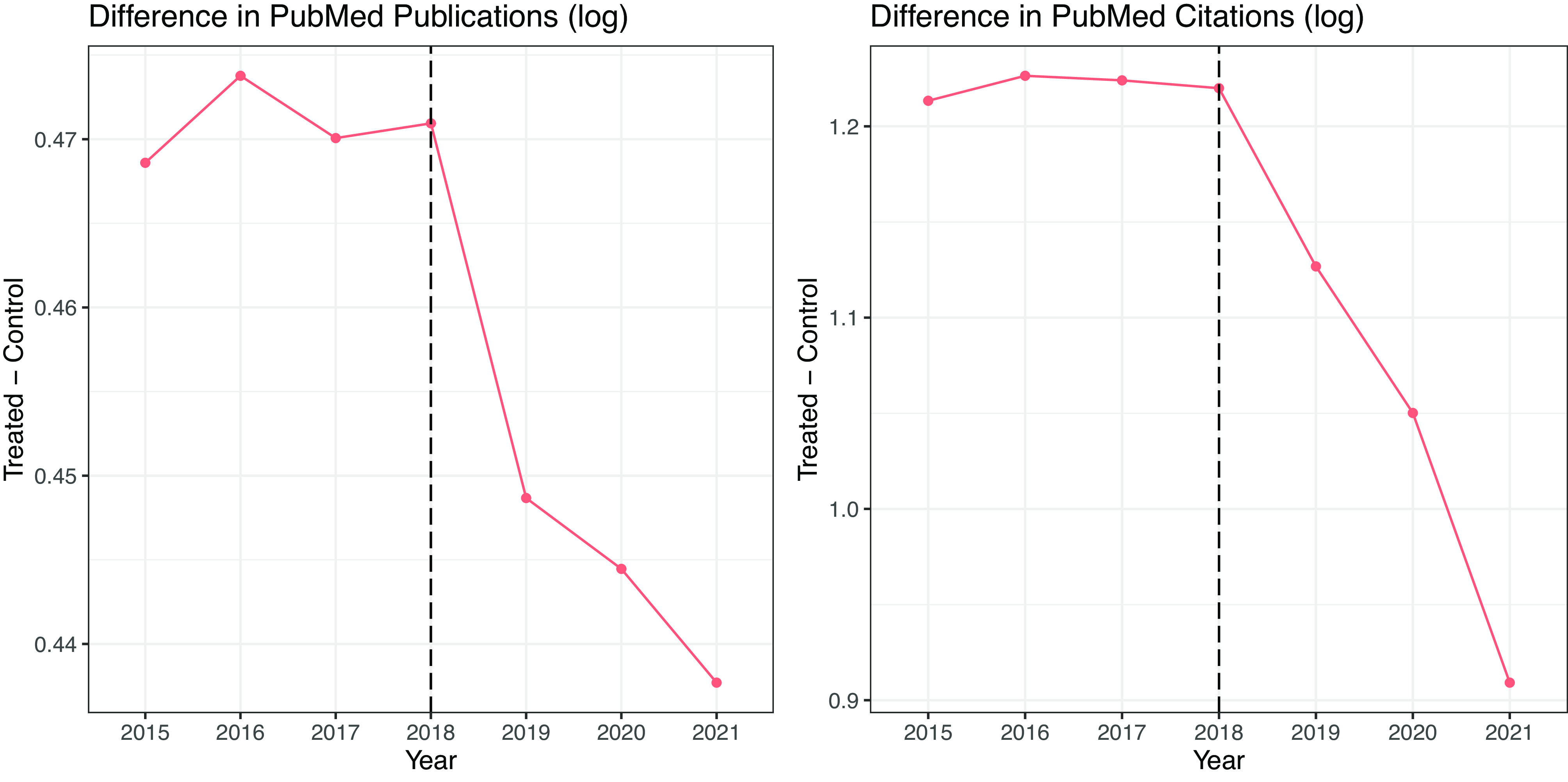
Differences in productivity between the treatment and control groups. *Note:* The figures present the differences in the logged number of PubMed publications and the logged number of PubMed citations between the treatment and control groups. We use log(1 + number of publications) and log(1 + number of citations) to facilitate interpretation.

To check the trends in the productivity of scientists in the treatment and control groups, we present the differences in the logged number of publications and the logged number of citations between the treatment and control groups by year in Fig. 2. As shown, the treated group is consistently more productive throughout the studied period. In other words, those with a collaboration history with scientists in China are among the more productive group of U.S. scientists. However, the productivity gap between the treated and the control appears to shrink after 2018, suggesting possible influence of political tensions. Note that the decline begins in 2019, before the pandemic in 2020.

Motivated by this evidence, we use a difference-in-differences (DID) design to investigate the causal impacts generated by the NIH investigations. Our specification is as follows:

[1] $$\begin{array}{c} Y_{i,t}=\beta 1\left\{TiesToChina_{i}\right\}*1\left\{Post_{t}\right\}+\alpha_{i}+\xi_{t}+X_{i}*\xi_{t}+\varepsilon_{i,t}, \end{array}$$

where $Y_{i,t}$ is the outcome of interest, such as the logged numbers of PubMed publications, total publications, and corresponding citations. (We present the distributions of these variables in *SI Appendix*, Fig. S3.) $1\{TiesToChina_{i}\}$ is a dummy indicating whether individual i belongs to the treated group; $1\{Post_{t}\}$ is a dummy that equals to 1 in the posttreatment periods and 0 otherwise. We also present the results when replacing $1\{TiesToChina_{i}\}$ with the share of China-collaborations in scientist i’s publications during 2010–2014 in *SI Appendix*.

$\alpha_{i}$ and $\xi_{t}$ stand for individual and year fixed effects, respectively. The individual fixed effects control for all time-invariant characteristics of a scientist such as gender and education background. The year fixed effects control for the factors that influence all scientists similarly such as the pandemic. Moreover, to further control for potentially different trends in productivity and personal background, we include four preinvestigation measures in $X_{i}$—one’s number of publications, citations, and NIH-supported publications during 2010–2014 and an indicator for being an Asian researcher—and allow for their impacts to vary over time by controlling for the interactions between scientist characteristics and year fixed effects($X_{i}*\xi_{t}$). We cluster the SE at the individual level to account for intertemporal correlation within each individual.

In addition to our main specification, we employ a reweighting approach, entropy balancing (30), to balance all covariates before running the regression and compare the estimates from our standard DID analysis. We further employ matching methods including propensity score matching and nearest neighbor matching (based on covariates) as a comparison. To check whether the treated group was in a different trend before the investigations, we complement our DID design with an event-study design and examine the impacts of the investigations year by year.

## 2. Results

### 2.1. Main Results: Quantity and Citations of Publications.

#### 2.1.1. Baseline estimates.

We present the DID estimates for our main outcomes in Table 2. Panel *A* shows the results for PubMed publications and citations. Column (1) uses the logged number and the vanilla two-way fixed effects model, without the controls. In Column (2), we control for the influences of each scientist’s preinvestigation productivity measures and their ethnicity. In Column (3), we report the estimate after conducting entropy balancing so that the baseline covariates are comparable between treated and control groups.^§§^ Column (4) transforms the outcome using inverse hyperbolic sine and Column (5) estimates the model using a Poisson likelihood. Columns (6)–(10) present the results for citations which capture both quantity and impact of the publications. All our results are similar if we use hyperbolic sine transformation to deal with observations of zeros (*SI Appendix*, Table S3) or if we estimate using a Poisson method (*SI Appendix*, Table S4).

**Table 2.** The impacts on productivity: main results

	(1)	(2)	(3)	(4)	(5)	(6)	(7)	(8)	(9)	(10)
Panel *A*	PubMed publications					PubMed citations				
Ties to China × Post	−0.027 (0.003)	−0.020 (0.003)	−0.021 (0.005)	−0.024 (0.004)	−0.038 (0.005)	−0.192 (0.007)	−0.099 (0.008)	−0.101 (0.012)	−0.095 (0.009)	−0.095 (0.015)
Pretreatment avg.	1.502	1.502	1.502	1.899	5.929	4.163	4.163	4.163	4.730	341.941
R2	0.732	0.732	0.783	0.723	0.549	0.660	0.662	0.706	0.652	0.777
Panel *B*	Non-PubMed publications					Non-PubMed citations				
Ties to China × Post	0.025 (0.003)	0.015 (0.004)	0.014 (0.008)	0.020 (0.004)	0.001 (0.010)	−0.079 (0.005)	−0.071 (0.006)	−0.057 (0.014)	−0.068 (0.007)	−0.023 (0.022)
Pretreatment avg.	0.981	0.981	0.981	1.247	3.454	1.401	1.401	1.401	1.665	41.819
R2	0.641	0.645	0.690	0.637	0.524	0.620	0.627	0.650	0.614	0.817
Panel *C*	Total publications					Total citations				
Ties to China × Post	−0.011 (0.003)	−0.008 (0.004)	−0.011 (0.006)	−0.010 (0.004)	−0.020 (0.005)	−0.180 (0.007)	−0.105 (0.008)	−0.105 (0.012)	−0.097 (0.009)	−0.082 (0.014)
Pretreatment avg.	1.878	1.878	1.878	2.346	9.383	4.470	4.470	4.470	5.070	383.760
R2	0.757	0.758	0.798	0.748	0.605	0.685	0.687	0.727	0.678	0.787
No. of obs.	792582	792582	792582	792582	792582	792582	792582	792582	792582	792582
Scholar FE	Y	Y	Y	Y	Y	Y	Y	Y	Y	Y
Year FE	Y	Y	Y	Y	Y	Y	Y	Y	Y	Y
Baseline covariates*Year FE		Y		Y	Y		Y		Y	Y
Entropy balancing			Y					Y		
Inverse hyperbolic sine				Y					Y	
Poisson					Y					Y

*Note:* For Columns (1)–(10), the models always control for scholar and year fixed effects. In Columns (2), (4), (5), (7), (9), and (10), we include the interactions between year dummies and four baseline covariates: 1) total number of publications in 2010–2014, 2) total citations in 2010–2014, 3) number of NIH-funded publications in 2010–2014, and 4) indicator for Asian researcher. Outcomes in columns (1)–(3) and (6)–(8) are log-transformed. Outcomes in columns (4) and (9) are transformed using inverse hyperbolic sine. Columns (3) and (6) use entropy balancing to balance all four covariates before running the regression. Columns (5) and (10) use Poisson regression. SE are clustered at the scholar level.

As shown in Columns (2)–(5) of Panel A, the number of PubMed publications of the treated scientists declined around 2.0–3.8%. However, the decline is more striking once the citations of the publications are considered: The decline becomes 9.5–10.1% compared with the control. These results reveal that the investigations may have affected not only the quantity but also the impact of publications of those who had collaboration histories with China.

Panel *B* presents the estimates for non-PubMed publications. In terms of quantity, we observe a minimal increase using our DID design and after balancing the covariates. However, once the impact of publications is considered, non-PubMed citations of the treated scientists declined by 2.3 to 7.1%. We consider all publications in Panel *C*. Again, the impact on the number of publications of the treated scientists is minimal but that on impact-adjusted productivity is sizable, with a decline of 10.5%.

**Fig. 3.**
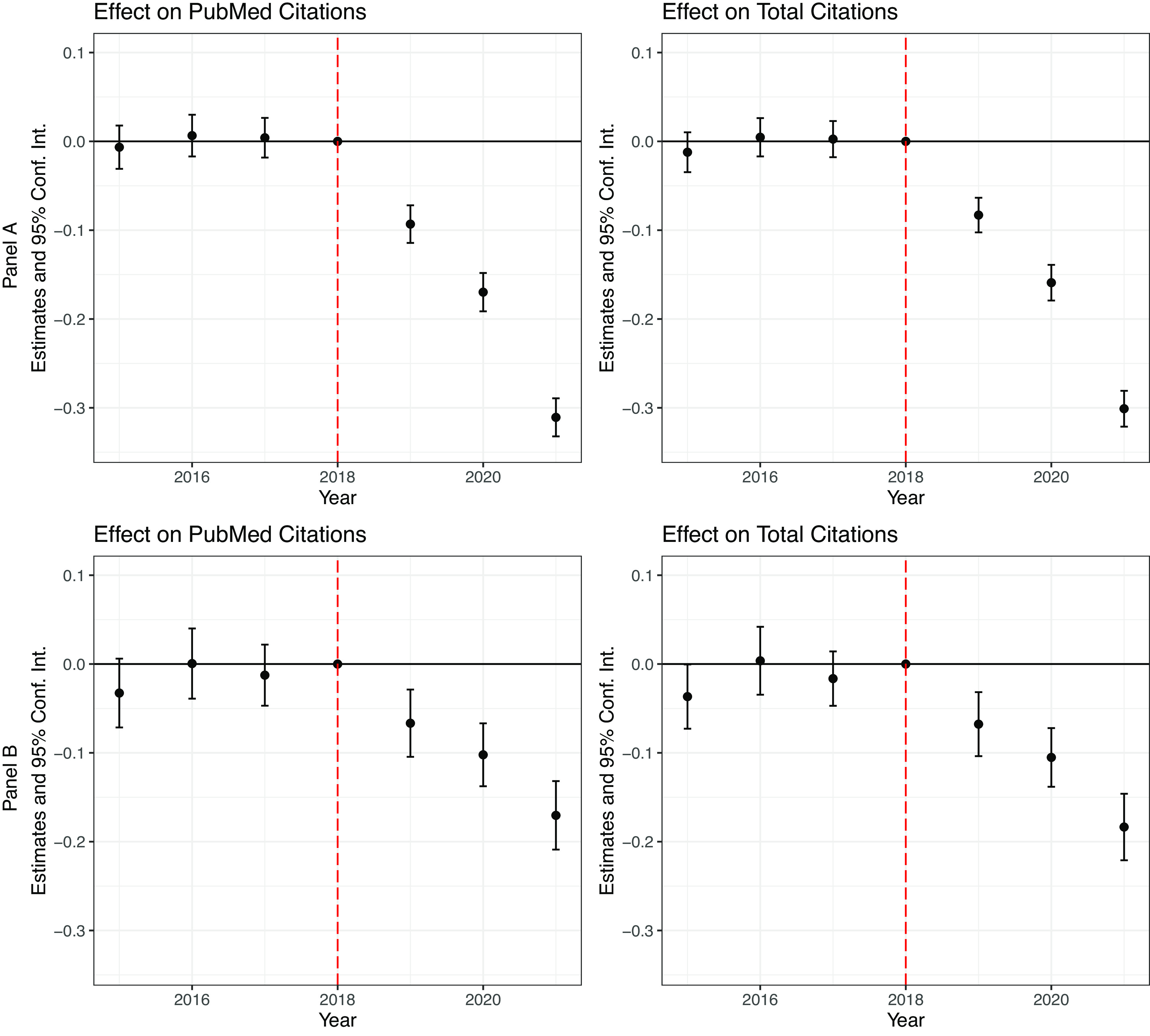
The impacts on productivity: results from event study. *Note:* Plots in this figure present the effect estimates of “leads and lags” of the treatment. Panel *A* presents the results controlling for scholar fixed effects and year fixed effects; Panel *B* presents the estimates using entropy balancing. Each segment represents the 95% CI of the estimate. The outcome in the left column is the logged number of citations for PubMed publications. In the right column, it is the logged number of citations for all publications.

Our identification assumption is that the productivity of scientists in our treated and control groups would be comparable without the NIH investigations. To check the validity of our assumption, we plot the year-by-year estimates in Fig. 3. Because our previous findings reveal that the citation of the publications is the main margin that gets affected, we focus on citations as the outcome. Panel *A* presents the estimates after only controlling for scholar fixed effects and yearly fixed effects whereas Panel B reports those after balancing the covariates. In either method, we find that the decline in productivity for the treated scientists occurred only after the investigations, suggesting that the pretrends concern is not critical for our findings.

#### 2.1.2. Additional results.

We employ alternative measures of research impact and observe similar negative impacts when using average citations or number of hit papers (defined based on relative citations in a given subfield, see *SI Appendix*, Table S5). However, we cannot distinguish of whether the decline in citations comes from a disinclination to cite a paper authored by the treated group or a decrease in quality, or both. Either way, our findings indicate a decline in research impact of treated scientists. In addition, we use journal ranking to proxy quality. We find that the negative impacts are similar when we focus on the publications on top-100 journals or those on other journals (*SI Appendix*, Table S6). Finally, we examine the share of COVID papers and find that excluding them does not affect our finding (*SI Appendix*, Tables S7 and S8).

Our baseline analysis uses a dummy variable to measure collaboration with China, which facilitates our comparison between the treated and the control. An extension is to measure the intensity of collaborations with scientists in China. Here, we measure the intensity by the logged number of publications collaborated with scientists in China during 2010–2014 (while controlling for logged total number of publications). As presented in *SI Appendix*, Table S9, our main finding holds when using this alternative measure of treatment except for one specification regarding non-PubMed publications. According to these estimates, scientists with a one-SD (0.68) more collaboration intensity with China experienced a 3.5% decline in terms of total citations after 2018.

We also examine the publications by funding sources and by collaboration types (*SI Appendix*, Table S10). The main takeaway is that the impacts are multidimensional. More specifically, we separate publications based on their funding sources. We find that the citation decline applies to both NIH-funded publications and non-NIH-funded publications and the former appears larger. Similarly, the citation decline applies to both China-funded publications and non-China-funded publications and the former is larger.^¶¶^ These results show that the adverse impacts on treated scientists are not limited to the publications funded by NIH or China. Instead, the productivity effect is reflected by different types of publications.

In *SI Appendix*, Table S11, we examine the publications by collaboration types—collaborations within the United States, collaborations with non-China countries, and collaborations with China. We note that the decline in China-collaborated publications in the treated group is not offset by an increase in collaborations with other countries. In terms of citations, we find all three types of collaborations were negatively affected for the treated group in comparison to the control group. These patterns suggest that the treated scientists may not have been able to use other types of collaborations to compensate for their loss in productivity.

In *SI Appendix*, Tables S12 and S13, we consider the potential influence of the investigations on migration. Although we cannot observe migration directly, we can proxy migration based on the country of affiliations in the scientists’ publications. Using this proxy, we find suggestive evidence that treated PIs are relatively more likely to move from the United States after 2018 compared to the control group. While this finding on migration outcomes aligns with ref. 24 and has important policy implications, we also confirm that our conclusions regarding publications and citations remain unaffected by migration. We find that the share of migrated scientists is small, and therefore the impact of migrated scientists is minimal for our finding on productivity.

### 2.2. Results by Institutions and Scientist Characteristics.

To better understand how prevalent our baseline finding is, we examine heterogeneous effects across institutions, scientist’s ethnicity, productivity, and career stage.

#### 2.2.1. By institutions.

**Fig. 4.**
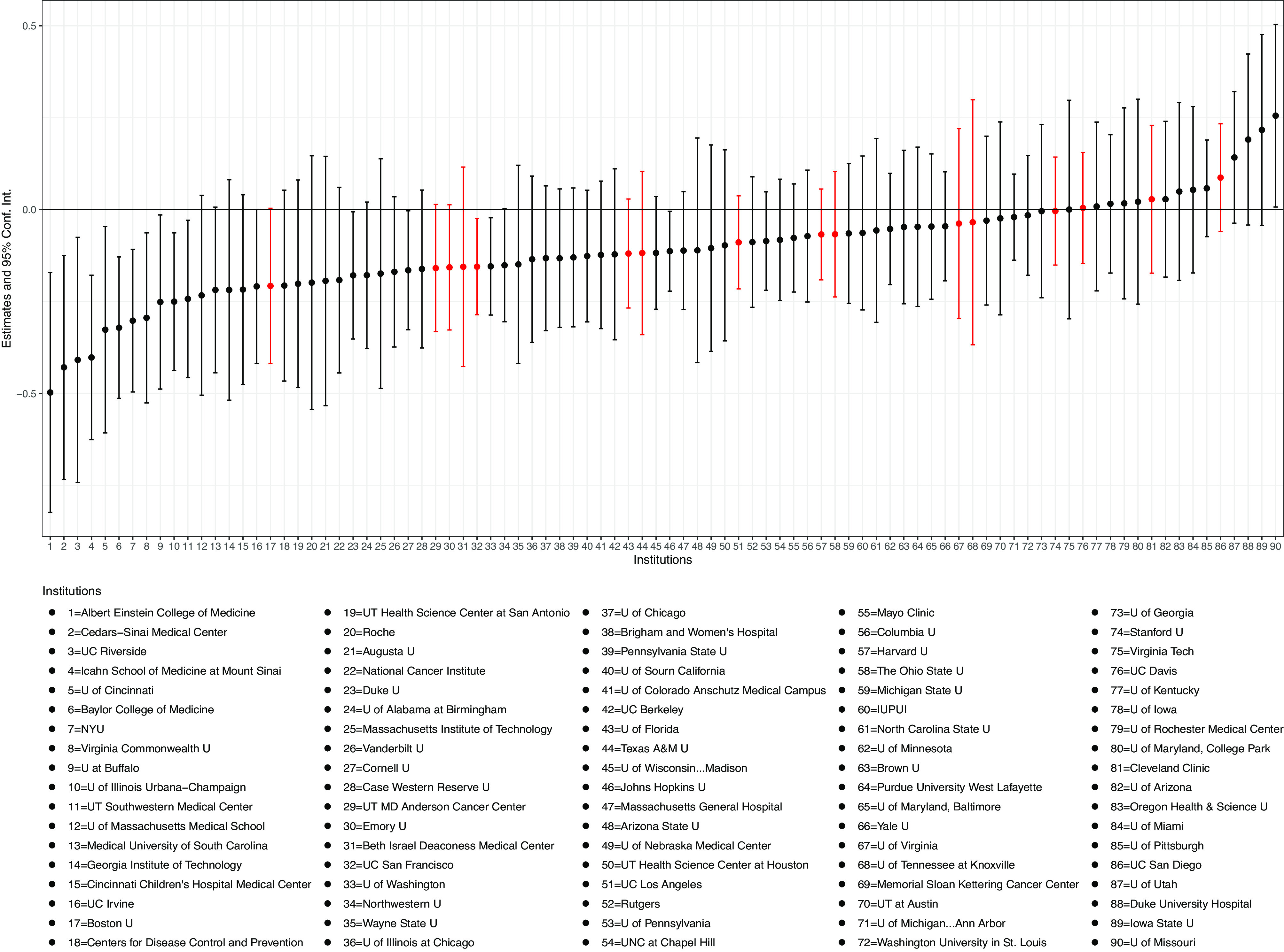
Heterogeneous treatment effects over institutions. *Note:* The figure presents the heterogeneity of treatment effects within the treated group across institutions in the sample that contain more than 100 scholars in both the treated group and the control group. Each point and error bar represent the estimated effect at a given institution and the corresponding 95% CI. Those in red represent institutions that are known to have scientist(s) investigated by the NIH.

We subset our sample by institution and estimate institution-specific treatment effects. For scientists with multiple institutions, we use their modal institution as their affiliation, which is defined as the institution in which a scientist published most of their work within the given period. In Fig. 4, we plot the heterogeneity of treatment effects for institutions in the sample that have more than 100 scholars in both the treated group and the control group. We find that the adverse effect applies to most of the institutions. In addition, we mark the institutions whose investigations were reported by the media in red. We identify institutions with public investigations using data from APA Justice and the *MIT Technology Review* (31). We do not find that the impacts on scholars in these institutions are different from those in other institutions. These results suggest that the impact is general and not institution-specific.

#### 2.2.2. By ethnicity.

Existing media reports on these investigations often highlight the role of ethnicity and focus on investigations at particular universities (32). Motivated by these discussions, we take a closer look at the ethnicity of scientists. Based on surnames, we predict the ethnicity of a scientist using the algorithm developed by Imai and Khanna (29) (see more details on the implementation of this algorithm in *SI Appendix*, section 1D).

With predicted ethnicity, we split the sample into Asian and non-Asian scientists and estimate a triple-difference design, as specified in the following equation:

[2] $$\begin{array}{cc} Y_{i,t} & =\beta_{1}1\left\{TiesToChina_{i}\right\}*1\left\{Post_{t}\right\}*1\left\{Asian_{i}\right\}\\ & \;+\beta_{2}1\left\{TiesToChina_{i}\right\}*1\left\{Post_{t}\right\}+\beta_{3}1\left\{Post_{t}\right\}\\ & \;*1\left\{Asian_{i}\right\}+\alpha_{i}+\xi_{t}+X_{i}*\xi_{t}+\varepsilon_{i,t}. \end{array}$$

On average, we find that both Asian and non-Asian scientists are adversely affected and the difference is small, as shown in Column (1) of Table 3. However, once we separate the publications to be those funded by NIH or not, we find that Asian scientists were more adversely affected in terms of NIH-funded publications, whereas the difference between Asian and non-Asian scientists is small but positive for non-NIH-funded publications [Columns (2) and (3)]. Moreover, we find that Asian scientists were also more adversely affected in terms of China-funded publications [Columns (4) and (5)].

**Table 3.** Heterogeneous treatment effects by ethnicity

	Citations by nature of publication				
	(1) All	(2) NIH-funded	(3) Non-NIH-funded	(4) China-funded	(5) Non-China-funded
Ties to China × Post × Asian	−0.008 (0.018)	−0.068 (0.018)	0.032 (0.018)	−0.222 (0.012)	0.001 (0.018)
Ties to China × Post	−0.103 (0.008)	−0.078 (0.009)	−0.071 (0.009)	−0.127 (0.005)	−0.089 (0.009)
Post × Asian	0.092 (0.013)	0.040 (0.012)	0.101 (0.013)	0.008 (0.003)	0.089 (0.013)
R2	0.687	0.662	0.642	0.570	0.680
No. of obs.	792582	792582	792582	792582	792582
Scholar FE	Y	Y	Y	Y	Y
Year FE	Y	Y	Y	Y	Y
Baseline covariates*Year FE	Y	Y	Y	Y	Y

*Note:* In all columns, outcomes are log-transformed and we control for scholar and year fixed effects, as well as the interactions of year dummies with the baseline covariates: 1) total number of publications in 2010–2014, 2) total citations in 2010–2014, and 3) number of NIH-funded publications in 2010–2014. SE are clustered at the scholar level.

#### 2.2.3. By productivity and career stage.

We further examine heterogeneity across pretreatment productivity and career stage. As reported in *SI Appendix*, Table S14, we find that negative impact is higher (and lower) for those with below-median productivity in relative (and absolute) terms. Moreover, the estimates are not significantly different for scientists by career stage (*SI Appendix*, Table S15), partly because scientists in our sample have already all established a record of international collaborations.

In sum, while there exists some heterogeneity across scientist characteristics, our takeaway is that the negative impact appears to be prevalent rather than restricted to a narrow group of scientists.

### 2.3. Results by Fields and Aggregate Implications.

We then decompose the effect by field of research. Given that the investigations were primarily at the NIH and focused mainly on U.S.–China collaborations, we expect that the findings are particularly relevant for the fields with more U.S.–China collaborations and the fields that receive a lot of funding from the NIH.^##^

#### 2.3.1. Estimates by fields.

We define research field using Dimensions metadata, which puts each publication into a “field of research” using the Australian and New Zealand Standard Research Classification. For each field, we create two measures using our publication data from 2010 to 2021. The first is the share of publications with NIH funding support in each field, the second the share of U.S.–China collaborations among total publications in each field. The top fields in terms of NIH funding in our data are biochemistry and cell biology, medical microbiology, and medical physiology, whereas the top fields in terms of U.S.–China collaborations are materials engineering, macromolecular and materials chemistry, and nanotechnology. We present these two measures by fields in *SI Appendix*, Table S16.

**Fig. 5.**
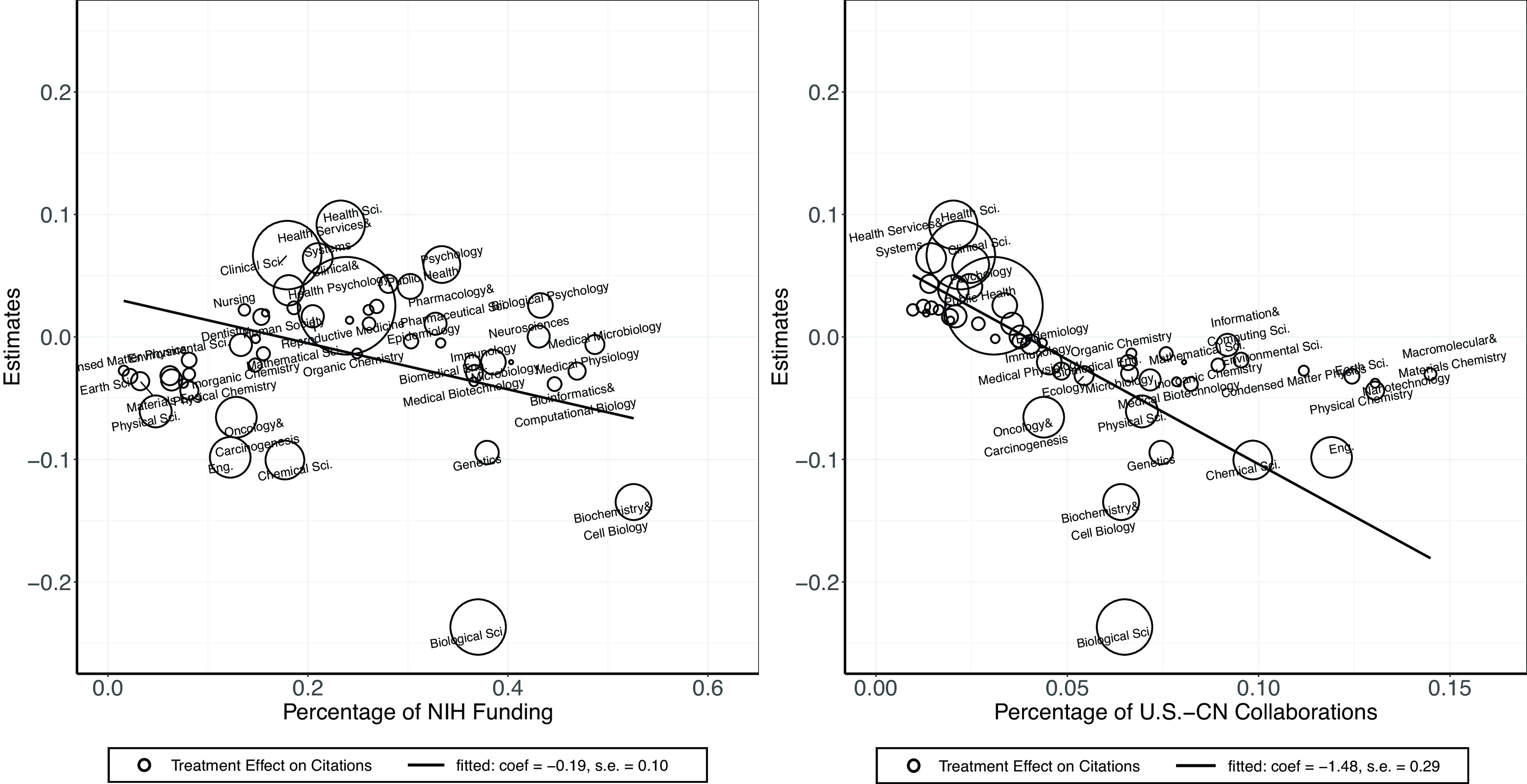
Citation estimates vs. NIH funding and U.S.–China collaborations. *Note:* Each bubble represents a field. The size of the bubbles is scaled by their number of publications in the data. The *Y*-axis is the estimated treatment effect on citations. The sample is restricted to fields with greater than 50,000 publications in our dataset.

We estimate the impacts of NIH investigations on citations by field (i.e., impact-adjusted productivity). Specifically, we subset our sample by field and estimate field-specific treatment effect on citations. We then correlate these estimates with the two measures above. As shown in Fig. 5, scientists with collaborations with institutions in China in the fields where NIH funding is more important experienced a larger decline relative to those in fields with less NIH funding. Specifically, a one-SD increase in the NIH funding (0.14) is associated with a 2.66 percentage point decline in the treatment effect. Similarly, scientists with collaborations with institutions in China in the fields where U.S.–China collaboration is more important experienced a larger decline relative to those in fields with less U.S.–China collaboration. The magnitude is even larger and more precisely estimated: a one-SD increase in the share of U.S.–China collaboration (0.04) is associated with a 6.0 percentage point decline in the treatment effect.

#### 2.3.2. Aggregate implications by fields.

Last, we provide a preliminary analysis of the effect of these investigations on the development of science in the United States and China more broadly. Did the NIH investigations matter for the development of science in the United States or China? It is challenging to provide a definite answer to this broad question. Nevertheless, the fact that we find that some fields were more affected by these investigations than others allows us to get some leverage on this question.

Conceptually, we would like to know how the progress of science by field in China and the United States in the last several years correlates with our findings by fields. Have fields that were most affected by the investigations according to our analysis slowed their progress in the United States and China in comparison to the rest of the world? Empirically, we measure the progress by fields in China and the United States relative to other countries using a difference-in-differences design. We first use Dimensions to collect data on the yearly number of publications by field for the top 50 countries (including China and the United States) in natural sciences research. We use the 2021 Nature Index (https://www.natureindex.com/annual-tables/2021/country/all) to select these 50 countries.

Mirroring our main design, we consider the research output during 2015–2018 as the pretreatment progress and the output during 2019–2021 as the posttreatment progress. Using the difference-in-differences design, for each field, we measure the increase or decrease in research output by field (f) for China and the United States, relative to the other 48 countries and the pretreatment period, estimated as follows:

[3] $$\begin{array}{cc} Y_{c,t} & =\beta_{f,CN}1\left\{CN\right\}*1\left\{Post_{t}\right\}+\alpha_{c}+\xi_{t}+\varepsilon_{c,t} \end{array}$$

[4] $$\begin{array}{cc} Y_{c,t} & =\beta_{f,US}1\left\{US\right\}*1\left\{Post_{t}\right\}+\alpha_{c}+\xi_{t}+\varepsilon_{c,t}, \end{array}$$

where $Y_{c,t}$ is the logged total number of publications in the field for country c in year t. To calculate the number of publications by field, country, and year, we queried Dimensions for total publications by country, year, and field. These totals thus reflect overall publications in the field, not just publication numbers by the scientists in our data described above. CN is an indicator for China and US is an indicator for the United States. We include country-level fixed effects ($\alpha_{c}$) and year fixed effects ($\xi_{t}$). For each field f, we then extract the estimate $\beta_{f,CN}$ and $\beta_{f,US}$ as an estimate of how China and the United States, respectively have fared in terms of productivity during 2019–2021 in comparison to the rest of the world.

**Fig. 6.**
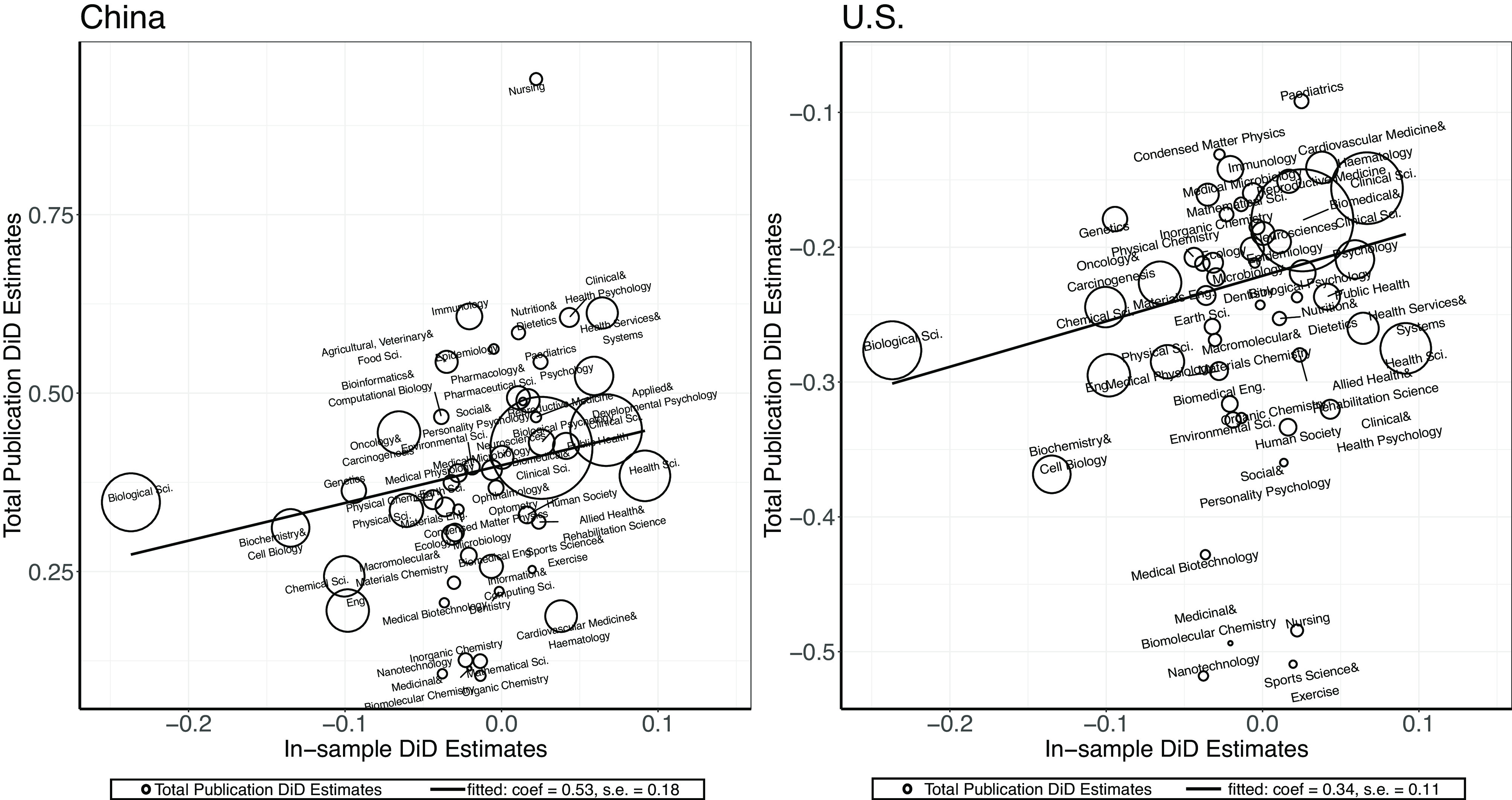
Citation estimates vs. progress by field in the United States and China. *Note:* Each dot represents a field. The *X*-axis is the estimated treatment effects on citations and the *Y*-axis is the estimated posttreatment research progress for China and the United States, relative to the other 48 countries and the pretreatment period. The figure shows the relationship between how much treated scientists’ publication citations in a field are impacted by the investigations (*x*-axis) and how much U.S. and China’s overall publications in that field are impacted. The sample is restricted to fields with greater than 50,000 publications in the data.

Fig. 6 shows the correlation between the estimates of the impact of NIH investigations on citations (*x*-axis) and the estimates on research progress based on the difference-in-differences design. This correlation can be interpreted as the elasticity of the scientific progress of the United States (and China) in response to the impacts of the investigations. As shown, there exists a positive correlation between our estimates and the increases in publications by field, indicating that the fields that are more affected by the U.S.–China political tensions have produced fewer new publications during 2019–2021 relative to the rest of the world. Notably, this positive relationship holds for both the United States and China. The slopes are 0.34 for the United States and 0.53 for China, suggesting that both countries appear to lose from these political tensions.

## 3. Discussion Based on Interviews

As a design complementary to our quantitative analyses, we have interviewed 12 scientists about their experience and perspectives. These interviews were approved by the UC San Diego Institutional Review Board. The majority of the scientists we talked to had previous, existing, or planned research collaborations with scientists in China. About half were of Chinese heritage, most were male, and all but two were senior rather than junior scholars. They covered five institutions and eight different fields of study, mostly in the life sciences and medicine, with a couple from the physical sciences. These interviews help us better understand underlying mechanisms for our finding that scholars with previous collaborations with China have seen a decrease in publications related to the life sciences and overall impact of publications following the NIH investigations.

Overwhelmingly, the scientists we interviewed felt affected by the investigations and recent U.S.–China tensions, and were reluctant to start new or continue existing projects with institutions in China. Most of the scientists reported that their research had been negatively affected by the investigations. For some scientists, the investigations had a direct effect on their research productivity. Two scientists we interviewed had had their NIH funding suspended for several years as a direct result of the investigations. This direct effect had a clear negative impact on their research, and in one case forced them to all but close their lab.

Even for those who were not directly affected by the investigations, some scientists saw a tradeoff between applying for U.S. government funding and continuing their international collaborations with institutions in China. These scientists reported that although they could technically continue their collaborations with U.S. government funding, doing so was risky as any mistake in reporting might be subject to intense scrutiny. Continuing collaborations with institutions in China, they reported, also had a new costly administrative overhead, including frequently consulting with their university’s administration to navigate constantly changing regulations about collaboration. They, therefore, felt they had to choose between access to U.S. research dollars and their collaborations with scientists in China.

This new reticence to continue collaborations with research groups in China was costly to productivity in several ways. Several scientists mentioned that the loss of collaboration with institutions in China meant loss of access to human capital, labs, and machines that were essential for their current work. Several scientists who we interviewed directly relied on equipment and labs in China as an input to their work. Many of the scientists reported using their collaborations as a way to recruit talented graduate students and postdocs.

Ceasing to collaborate with researchers in China often required U.S. researchers to change their research direction. Several mentioned that they were pursuing new research directions as a result of the policies. Two mentioned that they had felt that their best research had been conducted with their colleagues in China and they worried that their future work in the absence of these collaborations would be less impactful.

We found that scientists with Chinese heritage experienced this chilling effect more acutely than those without. The few scientists we interviewed who felt that their research had not been affected much by recent tensions were not of Chinese heritage. Several scientists we interviewed who were of Chinese heritage reported feeling under increased scrutiny because of their ethnicity.

Our quantitative and qualitative findings reveal that the scientists are affected in multiple dimensions. They also suggest that these investigations may have consequences unexpected by policy makers. For instance, we find a broad adverse effect on scientific productivity across institutions and fields, not just those related to national security. Moreover, as suggested by the comparison of scientific progress by fields, the investigations have aggregate implications that are important to be considered. Importantly, most of our interviewed scientists reported that they believe U.S.–China tensions are likely to last and thus have consequences in the long run. While the China Initiative has officially ended, funding agencies’ investigations of researchers are ongoing and universities’ policies with respect to collaborations with scientists in China are still in flux. We hope that our study serves as a step to understanding the consequences of the ongoing political tensions and opening up avenues for future research.

## 4. Materials and Methods

### 4.1. Data Construction.

In order to assess the impact of NIH investigations on the scientific output of U.S. scientists, we construct a dataset of U.S. scientists whose primary fields are in the medical and life sciences. To do so, we first query Dimensions to get the list of 1,440,402 PubMed publications in 2010–2014, for which at least one of the authors is based in the United States. We impose two restrictions on the scientists in our dataset: 1) each scientist has to have at least two PubMed publications in 2010–2014 for which they are the Principal Investigator (PI); and 2) at least one of their publications needs to have a U.S. affiliation. To determine the PI of each paper, we treat the last author of each paper as the PI for that paper, as per the convention in the life sciences. When information about corresponding authors is available in the data, we also include the corresponding authors as the PIs of the paper. The criterion (1) selects authors whose primary fields are more likely to be in the medical and life sciences and (2) focuses our attention on scientists who are based in the United States. Applying the restrictions results in a list of 208,647 scientists.

Based on the initial list of scientists, we query Dimensions to get all of the selected scientists’ publications (including non-PubMed publications) from 2015 to 2021. To ensure the scientists we study were still in the United States immediately before treatment, we further restrict that each scientist’s last publication prior to the beginning of the NIH investigations (August 20, 2018) shows they have a U.S. affiliation. This reduces the number of scientists to 192,493. Using affiliation data from Dimensions, we determine whether each paper included a U.S.–China collaboration, a U.S. collaboration with any country other than China, or included only authors from the United States. We also use Dimensions-provided data to keep track of other metadata such as the funding information, citation count, and research field of each paper for our analysis.

### 4.2. Validating Data Quality.

Because of the scale of the PubMed and Dimensions data and the algorithmic approach to coding authors, papers, and institutions that these databases use to produce them, the data inevitably have errors. To check the extent to which Dimensions data aligns with other existing datasets, we validate our data from Dimensions with data from Google Scholar, which is thought to be the most complete in terms of counting publication citations, but does not have an API for researcher access (33). In particular, we check whether our main outcomes of interest we use in this paper—authors’ publication record and citation counts—are comparable between the two sources.

To do so, we draw a random sample of 100 authors from our data. For these 100 authors, we are able to identify 54 authors who have Google Scholar profiles. For each matched author, we compare their number of publications in 2010–2020 based on Dimensions with that based on Google Scholar. We also compare citation counts for each author-year from the two data sources. We should note that Google Scholar includes information on working papers that have not been published.

Both measures are highly correlated between Dimensions and Google Scholar, with correlations around 0.82 (*SI Appendix*, Fig. S2). This gives us confidence that Dimensions data captures similar dynamics to other comparable data sources.

## Supplementary Material

Appendix 01 (PDF)

## Data Availability

Some study data available. All code used to produce the results are available. Due to copyright restrictions from Dimensions, raw data cannot be made available; however, aggregated data are made available to replicate the results in the paper at https://doi.org/10.7910/DVN/XUHAE1 (34).

## Footnotes

^*^See: https://www.justice.gov/nsd/information-about-department-justice-s-china-initiative-and-compilation-china-related

^†^See Dear Colleague Letter from NIH Director Francis Collins: https://www.insidehighered.com/sites/default/files/media/NIH%20Foreign%20Influence%20Letter%20to%20Grantees%2008-20-18.pdf. We summarize more information on the background of NIH investigations in *SI Appendix*, section 1A.

^‡^Lauer, Michael. “Foreign Interference in National Institutes of Health Funding and Grant Making Processes: A Summary of Findings From 2016 to 2021.” July 30, 2021. https://grants.nih.gov/grants/files/NIH-Foreign-Interference-Findings-2016-2018.pdf.

^§^There were a few cases where the Department of Justice investigated scientists with ties to China before the China Initiative began, for example Sherry Chen in 2014 and Xiaoxing Xi in 2015. However, the China Initiative and NIH Investigations in 2018 instituted a categorical shift in the number and breadth of the investigations.

^¶^We should note that Chinese science policy also experienced changes over time. Since the national leadership change in 2012, the Chinese science policy has been increasingly emphasizing “indigenous innovation,” whose influence on international collaborations remains to be understood (23). Since these changes do not take the form of a sharp shock in 2018 and are not as field-specific as the NIH investigations, it seems difficult to assume that our study reflects the influence of Chinese policies.

^#^Using 2014 as a cutoff allows us to examine pretrends (i.e., 2015–2018) for our differences-in-differences analysis. Our findings are robust to alternative cutoffs around 2014.

^‖^Based on our interviews, we think the effect materialized so quickly because many scientists knew soon after the NIH letter to universities in August 2018 that collaborations with China would be under particular scrutiny, and therefore some of their existing projects were impacted.

^**^In addition, to rule out an effect of COVID-19 research, we provide a robustness check in *SI Appendix*, section 2 where we remove all papers with titles containing the word “COVID.” We find that our results are unchanged.

^††^In our data, 130,072 U.S. scientists with international collaboration during 2010–2014 publish on average 6.39 papers per year, compared to 62,421 U.S. scientists without international collaboration during the same period, who publish on average 2.09 papers per year.

^‡‡^This paper was written using data obtained on October 23, 2022, from the API for Digital Science’s Dimensions platform, available at https://app.dimensions.ai. Total citations are measured by citations as of October 23, 2022. when we downloaded the data from Dimensions.

^§§^By design, the covariates are balanced using entropy balancing. We present estimates from propensity score matching and nearest neighbor matching (based on covariates) and related balance tests in *SI Appendix*, Tables S1 and S2. As shown, the estimates from different methods are similar.

^¶¶^We identify NIH funding using the funding information provided in Dimensions. China-funded publications are also estimated by Dimensions, which geolocates funders mentioned in academic papers. As described in *SI Appendix*, section 1A, mentions of funding from China in academic papers may have been a source of scrutiny by the NIH. This might be the reason that acknowledgment of funding from China in academic papers is more negatively affected.

^##^Field-specific effects were reflected in our interviews with scientists. Scientists who were in fields with high NIH-funding but low overall levels of U.S.–China collaboration, for example, public health and clinical sciences, felt much less pressure to stop their U.S.–China collaborations than those in fields with higher levels of U.S.–China collaboration, such as in chemistry and the biological sciences.
