# Supplementary material for: The impact of US–China tensions on US science: Evidence from the NIH investigations
Source: Proc Natl Acad Sci U S A. 2024 Apr 30;121(19):e2301436121. doi: 10.1073/pnas.2301436121 (PMC11087765; doi:10.1073/pnas.2301436121)
Supplement: Supplementary file 1 — Appendix 01 (PDF) [file pnas.2301436121.sapp.pdf]

Supporting Information for

The Impact of U.S.-China Tensions on U.S. Science: Evidence from the NIH Investigations

Ruixue Jia, Margaret E. Roberts, Ye Wang and Eddie Yang

Margaret E. Roberts
E-mail: meroberts@ucsd.edu

This PDF file includes:

Figs. S1 to S3
Tables S1 to S16
SI References

1. More on Background and Data Construction

A. Background of NIH Investigations. The NIH investigations began with a Dear Colleague letter in August of 2018 to many institutions of higher education.* As described in detail in (1), the NIH sent more detailed letters to approximately 100 institutions, with questions about 246 scientists. 81% of the scientists were from Asian descent and 90% of the cases involved resources or activities in China. Many of the allegations were that the scientists had not disclosed affiliations, collaborations, or funding from international sources. (See https://grants.nih.gov/sites/default/files/Foreign-Interference-12-9-22-report.pdf for NIH data on the investigations.) According to interviews conducted by (1), it appeared that the NIH had identified these individuals in part from the acknowledgement section of academic papers, which contained information about co-authors or funding from China. 42% of these investigated scientists were terminated from their job, and about 20% were banned from NIH funding.

B. Trends in Collaboration Patterns of Chinese Scientists. Figure 1 in the main text provides the changes in the collaboration patterns of U.S. PubMed publications. Here, Figure S1 shows the changes for Chinese PubMed publications. Similar to patterns found for the U.S. PubMed publications, the share of PubMed publications by scientists in China that are collaborations with U.S. scientists declined in recent years, although the decline seems to have started around 2016.

In addition, during its zenith, collaborations between the U.S. and China contribute to nearly 15% of Chinese publications in PubMed, whereas they constitute 8% of U.S. publications. This contrast underscores the greater significance of the U.S. as a collaborative partner for China compared to the reverse scenario.

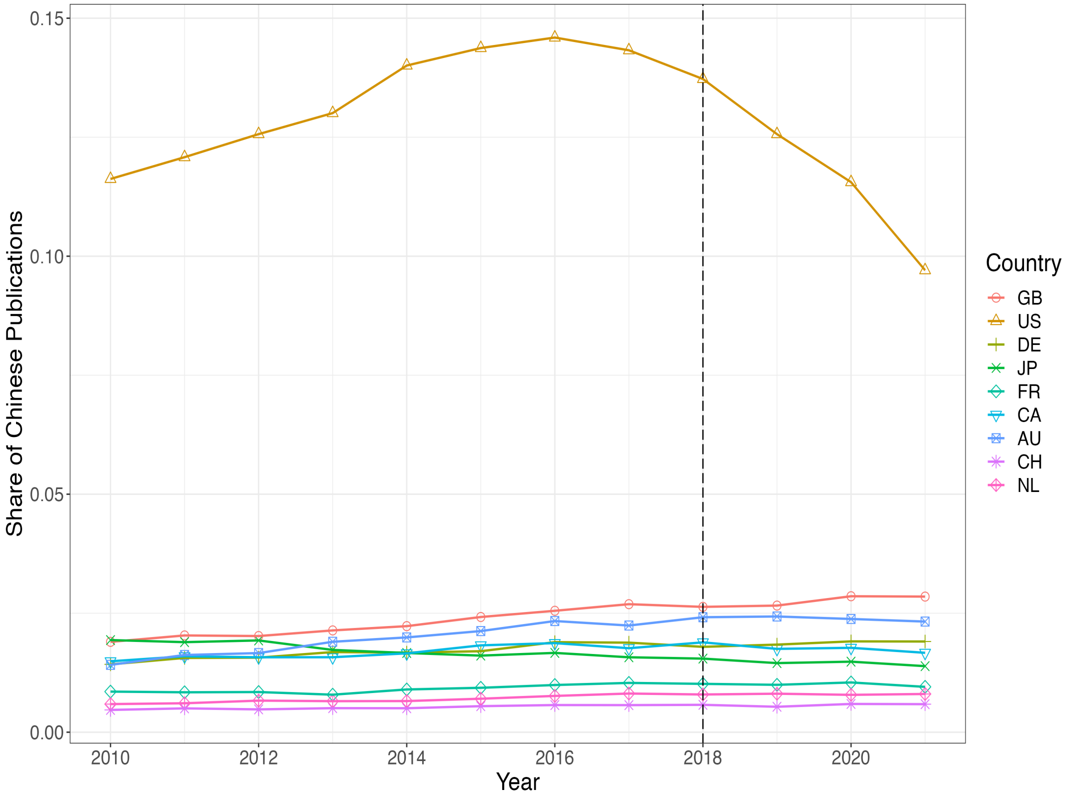

Fig. S1. Collaboration as Share of Total Chinese PubMed Publications

Note: The data is based on publications indexed by PubMed from Dimensions (see more on the data construction in Section 2 of the main text). Each line represents Chinese collaboration with a given country in PubMed publications as its share of total Chinese PubMed publications. Note that the data include all scientists in the Dimensions database, not just those included in the data we describe below.

* https://www.insidehighered.com/sites/default/files/media/NIH%20Foreign%20Influence%20Letter%20to%20Grantees%2008-20-18.pdf

C. Data Validation. For both publications and citations, we find high correlations between Dimensions data and Google Scholar data (collected March of 2022) for a sample of scientists, which gives us confidence in using Dimensions data. As shown in Figure S2 below, the correlations are around 0.82 for both measures.

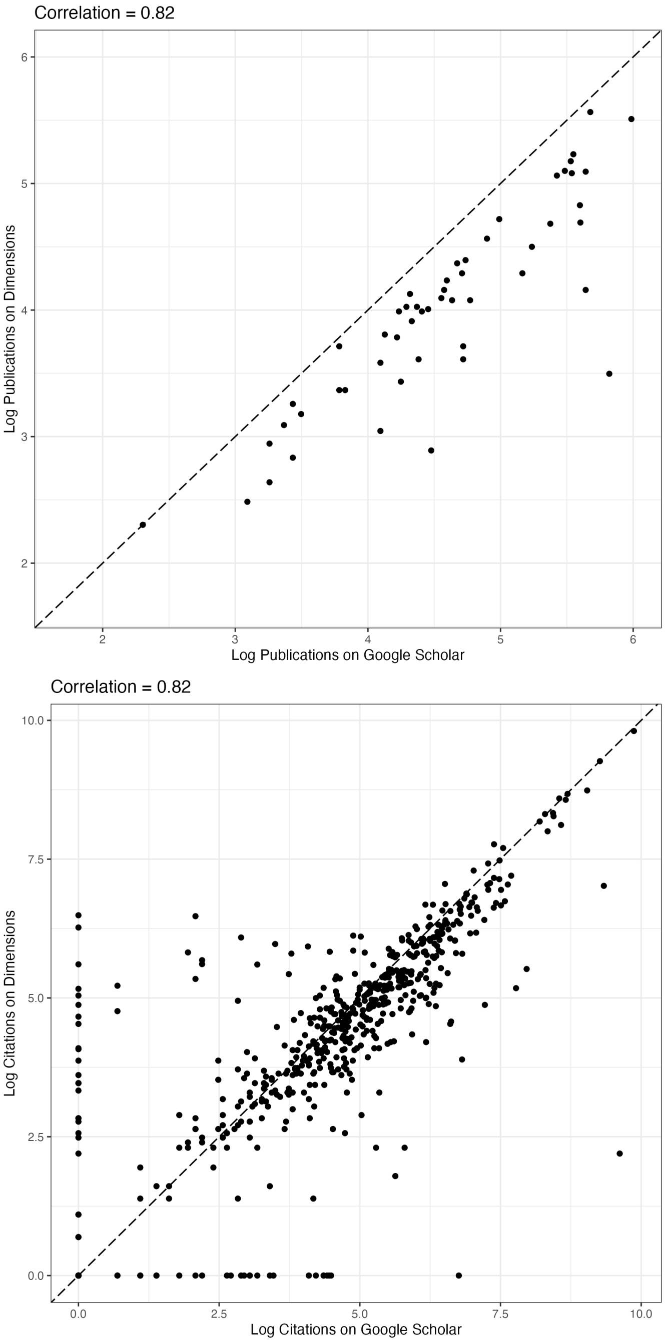

Fig. S2. Correlations of Data Between Dimensions and Google Scholar

Note: Each dot represents an author. The X-axis is logged number of publications (upper panel) and citations (lower panel)in 2010–2020 from Google Scholar and the Y-axis is logged number of publications and citations in 2010–2020 from Dimensions. Dashed line is the 45 degree line representing perfect correlation.

D. Predicting Asian Surnames. We predict the ethnicity of each scientist in our sample using the methodology developed by (2). The authors construct their training set by combining Census Bureau's Surname List with various information from voter registration records. They then use Bayes' rule to predict the posterior probability of each individual with given demographic information to belong to each of the five ethnic groups: White, Black, Hispanic, Asian and Other. We implement the method using the R package *wru*, which generates probability estimates for each surname in our sample. The most frequent surnames that are considered Asian by the method include *Wang* (578), *Chen* (447), and *Zhang* (400). It is worth noting that the method cannot distinguish Chinese surnames from other Asian surnames. Hence, the most common Asian surnames also include Korean surnames (e.g. *Kim*) or South Asian surnames (e.g. *Singh*).

2. Additional Results on Our Main Finding

A. Distribution of main outcome variables.

Fig. S3. Distribution of Main Outcome Variables

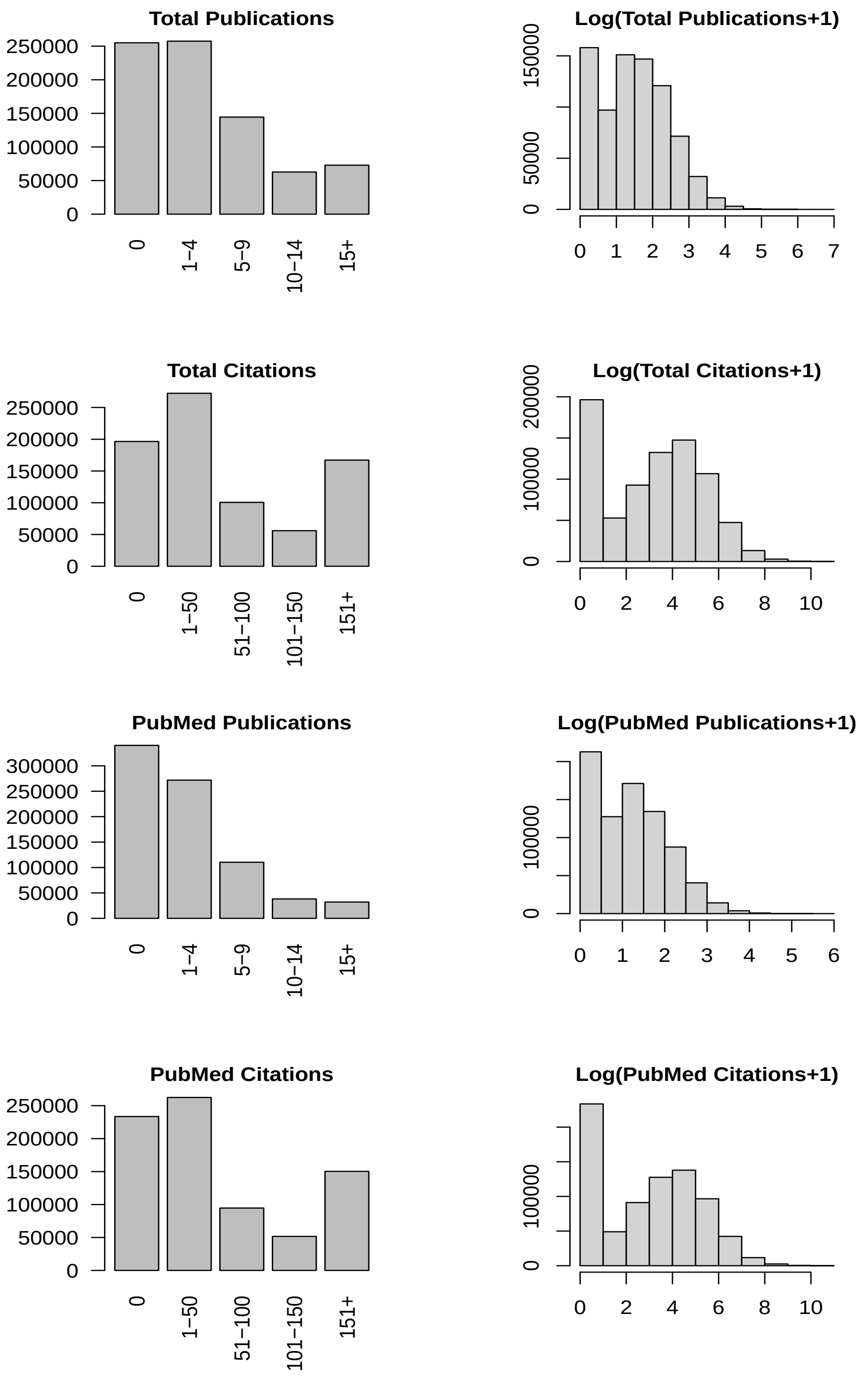

Note: These plots show the distribution of our main outcome variables in the analysis. The left panel presents the distribution of the original variables. The right panel shows the distribution of the logarithm transformation of the variables.

B. More Results on Balancing Strategies. We employ additional matching methods, including propensity score balancing and nearest neighbor matching (based on covariates), for robustness. Table S1 presents the balance tests across these methods, and Table S2 compares the results using different methods. As shown, the results are very similar across the three strategies. Compared with the other methods, entropy balancing has the advantages of not dropping observations.

Table S1. Balance Tests

	Treated Group	Control group			
	Raw Data	Raw Data	Entropy Balancing	Propensity Score Matching	Nearest Neighbor Matching
Total Publications	45.87	22.84	45.87	46.04	43.95
Total Citations	2774.09	925.87	2774.09	2760.38	2553.43
NIH Publications	14.29	6.36	14.29	13.88	13.61
Asian	0.29	0.12	0.29	0.28	0.28

Table S2. The Impacts on Productivity: Alternative Balancing Strategies

	(1)	(2)	(3)	(4)	(5)	(6)
**Panel A**	PubMed Publications			PubMed Citations		
Ties to China × Post	-0.021	-0.021	-0.023	-0.101	-0.121	-0.119
	(0.005)	(0.003)	(0.003)	(0.012)	(0.008)	(0.008)
Pre-treatment avg.	1.502	1.502	1.502	4.163	4.163	4.163
R2	0.783	0.735	0.735	0.706	0.664	0.662
No. of obs.	792582	621978	620326	792582	621978	620326
Scholar FE	Y	Y	Y	Y	Y	Y
Year FE	Y	Y	Y	Y	Y	Y
Entropy Balancing	Y			Y		
Propensity Score Matching		Y			Y	
Nearest Neighbor Matching			Y			Y
**Panel B**	Non-PubMed Publications			Non-PubMed Citations		
Ties to China × Post	0.014	0.022	0.020	-0.057	-0.065	-0.065
	(0.008)	(0.003)	(0.003)	(0.014)	(0.006)	(0.006)
Pre-treatment avg.	0.981	0.981	0.981	1.401	1.401	1.401
R2	0.690	0.647	0.647	0.650	0.634	0.634
No. of obs.	792582	621978	620326	792582	621978	620326
Scholar FE	Y	Y	Y	Y	Y	Y
Year FE	Y	Y	Y	Y	Y	Y
Entropy Balancing	Y			Y		
Propensity Score Matching		Y			Y	
Nearest Neighbor Matching			Y			Y
**Panel C**	Total Publications			Total Citations		
Ties to China × Post	-0.011	-0.006	-0.009	-0.105	-0.112	-0.112
	(0.006)	(0.004)	(0.004)	(0.012)	(0.007)	(0.007)
Pre-treatment avg.	1.878	1.878	1.878	4.470	4.470	4.470
R2	0.798	0.759	0.758	0.727	0.689	0.688
No. of obs.	792582	621978	620326	792582	621978	620326
Scholar FE	Y	Y	Y	Y	Y	Y
Year FE	Y	Y	Y	Y	Y	Y
Entropy Balancing	Y			Y		
Propensity Score Matching		Y			Y	
Nearest Neighbor Matching			Y			Y

Note: All outcomes are log-transformed, and the models always control for scholar and year fixed effects. Columns (1) and (4) present results using entropy balancing; Columns (2) and (5) present results using propensity score balancing; while Columns (3) and (6) present results using nearest neighbor matching. Standard errors are clustered at the scholar level.

C. Outcome with Inverse Hyperbolic Sine Transformation.

Table S3. Impacts on Productivity: Inverse Hyperbolic Sine Transformation

	(1)	(2)	(3)	(4)	(5)	(6)
**Panel A**	PubMed Publications			PubMed Citations		
Ties to China × Post	-0.032	-0.024	-0.025	-0.175	-0.095	-0.095
	(0.004)	(0.004)	(0.006)	(0.008)	(0.009)	(0.013)
Pre-treatment avg.	1.899	1.899	1.899	4.730	4.730	4.730
R2	0.722	0.723	0.772	0.651	0.652	0.696
No. of obs.	792582	792582	792582	792582	792582	792582
Scholar FE	Y	Y	Y	Y	Y	Y
Year FE	Y	Y	Y	Y	Y	Y
Baseline Covariates*Year FE		Y			Y	
Entropy Balancing			Y			Y
**Panel B**	Non-PubMed Publications			Non-PubMed Citations		
Ties to China × Post	0.030	0.020	0.017	-0.075	-0.068	-0.053
	(0.004)	(0.004)	(0.009)	(0.006)	(0.007)	(0.016)
Pre-treatment avg.	1.247	1.247	1.247	1.665	1.665	1.665
R2	0.633	0.637	0.681	0.607	0.614	0.638
No. of obs.	792582	792582	792582	792582	792582	792582
Scholar FE	Y	Y	Y	Y	Y	Y
Year FE	Y	Y	Y	Y	Y	Y
Baseline Covariates*Year FE		Y			Y	
Entropy Balancing			Y			Y
**Panel C**	Total Publications			Total Citations		
Ties to China × Post	-0.011	-0.010	-0.013	-0.157	-0.097	-0.097
	(0.004)	(0.004)	(0.007)	(0.008)	(0.009)	(0.012)
Pre-treatment avg.	2.346	2.346	2.346	5.070	5.070	5.070
R2	0.748	0.748	0.787	0.677	0.678	0.718
No. of obs.	792582	792582	792582	792582	792582	792582
Scholar FE	Y	Y	Y	Y	Y	Y
Year FE	Y	Y	Y	Y	Y	Y
Baseline Covariates*Year FE		Y			Y	
Entropy Balancing			Y			Y

Note: All outcomes are transformed with inverse hyperbolic sine. For Columns (1) to (6), the models always control for scholar and year fixed effects. In Columns (2) and (5), we include the interactions between year dummies and four baseline covariates: 1) total number of publications in 2010-2014, 2) total citations in 2010-2014, 3) number of NIH-funded publications in 2010-2014, and 4) indicator for Asian researcher. In Columns (3) and (6), we use entropy balancing to balance all four covariates before running the regression. Standard errors are clustered at the scholar level.

D. Results Using Poisson Likelihood.

Table S4. Impacts on Productivity: Poisson Likelihood

	(1)	(2)	(3)	(4)	(5)	(6)
**Panel A**	PubMed Publications			PubMed Citations		
Ties to China × Post	-0.035	-0.038	-0.038	-0.113	-0.095	-0.152
	(0.005)	(0.005)	(0.019)	(0.014)	(0.015)	(0.078)
Pre-treatment avg.	5.929	5.929	5.929	341.941	341.941	341.941
R2	0.549	0.549	0.517	0.776	0.777	0.420
No. of obs.	792582	792582	792582	792582	792582	792582
Scholar FE	Y	Y	Y	Y	Y	Y
Year FE	Y	Y	Y	Y	Y	Y
Baseline Covariates*Year FE		Y			Y	
Entropy Balancing			Y			Y
**Panel B**	Non-PubMed Publications			Non-PubMed Citations		
Ties to China × Post	-0.005	0.001	-0.032	0.032	-0.023	0.110
	(0.008)	(0.010)	(0.060)	(0.019)	(0.022)	(0.082)
Pre-treatment avg.	3.454	3.454	3.454	41.819	41.819	41.819
R2	0.523	0.524	0.494	0.815	0.817	0.841
No. of obs.	792582	792582	792582	792582	792582	792582
Scholar FE	Y	Y	Y	Y	Y	Y
Year FE	Y	Y	Y	Y	Y	Y
Baseline Covariates*Year FE		Y			Y	
Entropy Balancing			Y			Y
**Panel C**	Total Publications			Total Citations		
Ties to China × Post	-0.020	-0.020	-0.031	-0.091	-0.082	-0.122
	(0.005)	(0.005)	(0.025)	(0.013)	(0.014)	(0.073)
Pre-treatment avg.	9.383	9.383	9.383	383.760	383.760	383.760
R2	0.605	0.605	0.548	0.786	0.787	0.496
No. of obs.	792582	792582	792582	792582	792582	792582
Scholar FE	Y	Y	Y	Y	Y	Y
Year FE	Y	Y	Y	Y	Y	Y
Baseline Covariates*Year FE		Y			Y	
Entropy Balancing			Y			Y

Note: All models are estimated using a Poisson likelihood. For Columns (1) to (6), the models always control for scholar and year fixed effects. In Columns (2) and (5), we include the interactions between year dummies and four baseline covariates: 1) total number of publications in 2010-2014, 2) total citations in 2010-2014, 3) number of NIH-funded publications in 2010-2014, and 4) indicator for Asian researcher. In Columns (3) and (6), we use entropy balancing to balance all four covariates before running the regression. Standard errors are clustered at the scholar level.

E. Alternative Metrics of Productivity. We consider four alternative metrics of productivity. The first and second refer to the average citations of all the papers or all the PubMed papers published by scholar i in year t. The third one captures the influence of a publication based on field citation ratio provided by Dimensions.[†] Based on this ratio, we further define a paper as a hit paper if its field citation ratio is above the 95% percentile of the variable in our sample (24). As shown in Table S5, all these outcomes are negatively affected by the treatment, implying that our main finding is robust to different metrics of productivity.

Table S5. The Impacts on Productivity: Alternative Outcome Variables

	(1)	(2)	(3)	(4)	(5)	(6)
**Panel A**	Avg. Citations			Avg. PubMed Citations		
Ties to China × Post	-0.143	-0.089	-0.088	-0.145	-0.075	-0.075
	(0.005)	(0.005)	(0.010)	(0.005)	(0.006)	(0.010)
Pre-treatment avg.	2.825	2.825	2.825	2.584	2.584	2.584
R2	0.548	0.550	0.583	0.527	0.530	0.568
No. of obs.	792582	792582	792582	792582	792582	792582
Scholar FE	Y	Y	Y	Y	Y	Y
Year FE	Y	Y	Y	Y	Y	Y
Baseline Covariates*Year FE		Y			Y	
Entropy Balancing			Y			Y
**Panel B**	Avg. Relative Citations			No. of Hit Papers		
Ties to China × Post	-0.137	-0.062	-0.059	-0.078	-0.013	-0.016
	(0.003)	(0.004)	(0.009)	(0.002)	(0.002)	(0.006)
Pre-treatment avg.	1.554	1.554	1.554	0.278	0.278	0.278
R2	0.503	0.518	0.553	0.426	0.472	0.493
No. of obs.	792582	792582	792582	792582	792582	792582
Scholar FE	Y	Y	Y	Y	Y	Y
Year FE	Y	Y	Y	Y	Y	Y
Baseline Covariates*Year FE		Y			Y	
Entropy Balancing			Y			Y

Note: All outcomes are log-transformed. For Columns (1) to (6), the models always control for scholar and year fixed effects. In Columns (2) and (5), we include the interactions between year dummies and four baseline covariates: 1) total number of publications in 2010-2014, 2) total citations in 2010-2014, 3) number of NIH-funded publications in 2010-2014, and 4) indicator for Asian researcher. In Columns (3) and (6), we use entropy balancing to balance all four covariates before running the regression. Standard errors are clustered at the scholar level.

[†] For each paper, field citation ratio is calculated by "dividing the number of citations a paper has received by the average number received by documents published in the same year and in the same Fields of Research (FoR) category."

F. Effects by Journal Rankings.

Table S6. Effects on Productivity by Journal Rankings

	(1)	(2)	(3)	(4)	(5)	(6)
**Panel A**	Top-100 Journal Publications			Top-100 Journal Citations		
Ties to China × Post	-0.022	-0.007	-0.008	-0.189	-0.080	-0.079
	(0.002)	(0.002)	(0.005)	(0.007)	(0.007)	(0.029)
Pre-treatment avg.	0.233	0.233	0.233	0.956	0.956	0.956
R2	0.613	0.615	0.670	0.438	0.444	0.483
No. of obs.	792582	792582	792582	792582	792582	792582
Scholar FE	Y	Y	Y	Y	Y	Y
Year FE	Y	Y	Y	Y	Y	Y
Baseline Covariates*Year FE		Y			Y	
Entropy Balancing			Y			Y
**Panel B**	Non-Top-100 Journal Publications			Non-Top-100 Journal Citations		
Ties to China × Post	-0.006	-0.007	-0.010	-0.165	-0.095	-0.098
	(0.003)	(0.004)	(0.007)	(0.007)	(0.008)	(0.013)
Pre-treatment avg.	1.837	1.837	1.837	4.326	4.326	4.326
R2	0.750	0.750	0.791	0.676	0.677	0.717
No. of obs.	792582	792582	792582	792582	792582	792582
Scholar FE	Y	Y	Y	Y	Y	Y
Year FE	Y	Y	Y	Y	Y	Y
Baseline Covariates*Year FE		Y			Y	
Entropy Balancing			Y			Y

Note: All outcomes are log-transformed. For Columns (1) to (6), the models always control for scholar and year fixed effects. In Columns (2) and (5), we include the interactions between year dummies and four baseline covariates: 1) total number of publications in 2010-2014, 2) total citations in 2010-2014, 3) number of NIH-funded publications in 2010-2014, and 4) indicator for Asian researcher. In Columns (3) and (6), we use entropy balancing to balance all four covariates before running the regression. Standard errors are clustered at the scholar level.

G. COVID Publications. Given the significance of the COVID pandemic, it is important to understand how that may impact the research of the scientists in our sample. Table S7 breaks down the percentage of COVID publications, out of all publications, for the treatment and control groups in 2020 and 2021. "COVID publications" are defined as those for which the titles contain either "COVID" or "coronavirus". As Table S7 shows, COVID publications account for a small percentage of the total number of publications of both the treatment and control groups.

Table S7. Percentage of COVID Publications by Group

	Treated	Control
2020	3.6%	4.3%
2021	3.9%	5.2%

Because there was essentially no COVID publication prior to 2020, we cannot use COVID publications as an outcome. To check the influence of COVID publications on the main results, we remove all COVID publications from the publication (and associated citation) records of the scientists in out sample and report the main results in Table S8. Removing COVID publications has little impact on the main results and does not change our substantive conclusions.

Table S8. The Impacts on Productivity: Excluding COVID Papers

	(1)	(2)	(3)	(4)	(5)	(6)
**Panel A**	PubMed Publications			PubMed Citations		
Ties to China × Post	-0.026	-0.015	-0.015	-0.186	-0.083	-0.082
	(0.003)	(0.003)	(0.005)	(0.007)	(0.008)	(0.012)
Pre-treatment avg.	1.502	1.502	1.502	4.163	4.163	4.163
R2	0.732	0.732	0.783	0.663	0.665	0.709
No. of obs.	792582	792582	792582	792582	792582	792582
Scholar FE	Y	Y	Y	Y	Y	Y
Year FE	Y	Y	Y	Y	Y	Y
Baseline Covariates*Year FE		Y			Y	
Entropy Balancing			Y			Y
**Panel B**	Non-PubMed Publications			Non-PubMed Citations		
Ties to China × Post	0.025	0.017	0.016	-0.082	-0.068	-0.055
	(0.003)	(0.004)	(0.008)	(0.005)	(0.006)	(0.013)
Pre-treatment avg.	0.981	0.981	0.981	1.401	1.401	1.401
R2	0.641	0.646	0.690	0.623	0.631	0.654
No. of obs.	792582	792582	792582	792582	792582	792582
Scholar FE	Y	Y	Y	Y	Y	Y
Year FE	Y	Y	Y	Y	Y	Y
Baseline Covariates*Year FE		Y			Y	
Entropy Balancing			Y			Y
**Panel C**	Total Publications			Total Citations		
Ties to China × Post	-0.008	-0.004	-0.006	-0.172	-0.088	-0.084
	(0.003)	(0.004)	(0.006)	(0.007)	(0.008)	(0.012)
Pre-treatment avg.	1.878	1.878	1.878	4.470	4.470	4.470
R2	0.758	0.759	0.799	0.689	0.691	0.731
No. of obs.	792582	792582	792582	792582	792582	792582
Scholar FE	Y	Y	Y	Y	Y	Y
Year FE	Y	Y	Y	Y	Y	Y
Baseline Covariates*Year FE		Y			Y	
Entropy Balancing			Y			Y

Note: All outcomes are log-transformed. For Columns (1) to (6), the models always control for scholar and year fixed effects. In Columns (2) and (5), we include the interactions between year dummies and four baseline covariates: 1) total number of publications in 2010-2014, 2) total citations in 2010-2014, 3) number of NIH-funded publications in 2010-2014, and 4) indicator for Asian researcher. In Columns (3) and (6), we use entropy balancing to balance all four covariates before running the regression. Standard errors are clustered at the scholar level.

H. Estimates Based on Treatment Intensity.

Table S9. The Impacts on Productivity: Treatment Intensity

	(1)	(2)	(3)	(4)
**Panel A**	PubMed Publications		PubMed Citations	
Log No. of China Collab. × Post	-0.018	-0.013	-0.122	-0.044
	(0.002)	(0.002)	(0.005)	(0.006)
R2	0.732	0.732	0.660	0.662
No. of obs.	792582	792582	792582	792582
Scholar FE	Y	Y	Y	Y
Year FE	Y	Y	Y	Y
Baseline Covariates*Year FE		Y		Y
**Panel B**	Non-PubMed Publications		Non-PubMed Citations	
Log No. of China Collab. × Post	0.014	0.012	-0.068	-0.055
	(0.002)	(0.003)	(0.004)	(0.005)
R2	0.641	0.645	0.620	0.627
No. of obs.	792582	792582	792582	792582
Scholar FE	Y	Y	Y	Y
Year FE	Y	Y	Y	Y
Baseline Covariates*Year FE		Y		Y
**Panel C**	Total Publications		Total Citations	
Log No. of China Collab. × Post	-0.009	-0.005	-0.113	-0.051
	(0.002)	(0.003)	(0.004)	(0.005)
R2	0.757	0.758	0.685	0.687
No. of obs.	792582	792582	792582	792582
Scholar FE	Y	Y	Y	Y
Year FE	Y	Y	Y	Y
Baseline Covariates*Year FE		Y		Y

Note: All outcomes are log-transformed. Treatment is the logged numbers of publications that are collaborations with Chinese scientists during 2010-2014. For Columns (1) to (4), the models always control for scholar and year fixed effects. In Columns (2) and (4), we include the interactions between year dummies and four baseline covariates: 1) total number of publications in 2010-2014, 2) total citations in 2010-2014, 3) number of NIH-funded publications in 2010-2014, and 4) indicator for Asian researcher. Standard errors are clustered at the scholar level.

I. Citation Effects by Funding Sources.

Table S10. Effects on Citations by Funding Sources

	Citation Effects by Nature of Publication				
	All	NIH-Funded	Non NIH-Funded	China-Funded	Non China-Funded
Ties to China × Post	-0.105	-0.093	-0.064	-0.174	-0.089
	(0.008)	(0.008)	(0.008)	(0.005)	(0.008)
Pre-treatment avg.	4.470	2.678	3.252	0.781	4.339
R2	0.687	0.662	0.642	0.570	0.680
No. of obs.	792582	792582	792582	792582	792582
Scholar FE	Y	Y	Y	Y	Y
Year FE	Y	Y	Y	Y	Y
Baseline Covariates*Year	Y	Y	Y	Y	Y

Note: In all columns, outcomes are log-transformed and we control for scholar and year fixed effects, as well as the interactions of year dummies with the baseline covariates: 1) total number of publications in 2010-2014, 2) total citations in 2010-2014, 3) number of NIH-funded publications in 2010-2014, and 4) indicator for being Asian researcher. Standard errors are clustered at the scholar level.

J. Effects by Collaboration Types.

Table S11. The Impacts on Productivity: Collaboration Types

	Effects by Nature of Publication					
	U.S. Publications	Intl (Non-China) Publications	China-Collab Publications	U.S. Citations	Intl (Non-China) Citations	China-Collab Citations
Ties to China × Post	0.011	-0.013	-0.058	-0.030	-0.116	-0.456
	(0.004)	(0.003)	(0.002)	(0.008)	(0.009)	(0.007)
Pre-treatment avg.	1.491	0.795	0.401	3.563	2.469	1.404
R2	0.716	0.636	0.681	0.627	0.530	0.565
No. of obs.	792582	792582	792582	792582	792582	792582
Scholar FE	Y	Y	Y	Y	Y	Y
Year FE	Y	Y	Y	Y	Y	Y
Baseline Covariates*Year FE	Y	Y	Y	Y	Y	Y

Note: In all columns, outcomes are log-transformed and we control for scholar and year fixed effects, as well as the interactions of year dummies with the baseline covariates: 1) total number of publications in 2010-2014, 2) total citations in 2010-2014, 3) number of NIH-funded publications in 2010-2014, and 4) indicator for being Asian researcher. Standard errors are clustered at the scholar level.

K. Consdiering Migration. In this section, we explore the potential effects of migration on our analysis of scientific productivity. Our baseline analysis does not consider migration and counts the publications of scientists regardless of their migration decisions. Now, we delve into how migration might influence our findings.

While we cannot observe migration directly, we consider a proxy for migration based on the country of affiliations in the publications. To ensure the reliability of our findings, we employ different migration thresholds, assuming that a scientist has "migrated" to another country if more than 50% or 75% of their publications are affiliated with non-U.S. countries only. Using these criteria, we document the proportions of migration for both our treated and control groups of scientists in Table S12 before and after the onset of the investigations.

Under the 50% threshold, the probabilities of migration for treated and control principal investigators (PIs) were 0.97% and 0.95%, respectively, before 2018. However, after 2018, these probabilities increased to 1.88% for treated PIs and 1.32% for the control group. This suggests that treated PIs might be relatively more likely to move from the United States after 2018 compared to the control group.

While this finding on migration outcomes aligns with (3) and has important policy implications, we can confirm that our conclusions regarding publications and citations remain unaffected by migration. Since the share of migrated scientists is small, the impact of migrated scientists is minimal for our finding on productivity. Table S13 shows the main results hold if we exclude those with more than 50% of publications affiliated with non-U.S. countries only.

Table S12. Migration Statistics

	Before Treatment		After Treatment	
Non-US publications	50%	75%	50%	75%
Treated	340 (0.97%)	106 (0.30%)	660 (1.88%)	372 (1.06%)
Control	738 (0.95%)	224 (0.29%)	1032 (1.32%)	700 (0.90%)

Note: Numbers in brackets show the proportions of migrated scientists with respect to the total number of scientists of each group.

Table S13. The Impacts on Productivity: Excluding Migrated Scientists

	(1)	(2)	(3)	(4)	(5)	(6)
**Panel A**	PubMed Publications			PubMed Citations		
Ties to China × Post	-0.028	-0.021	-0.022	-0.194	-0.101	-0.104
	(0.003)	(0.003)	(0.005)	(0.007)	(0.008)	(0.012)
Pre-treatment avg.	1.501	1.501	1.501	4.164	4.164	4.164
R2	0.731	0.731	0.782	0.660	0.661	0.706
No. of obs.	776062	776062	776062	776062	776062	776062
Scholar FE	Y	Y	Y	Y	Y	Y
Year FE	Y	Y	Y	Y	Y	Y
Baseline Covariates*Year FE		Y			Y	
Entropy Balancing			Y			Y
**Panel B**	Non-PubMed Publications			Non-PubMed Citations		
Ties to China × Post	0.023	0.014	0.010	-0.081	-0.073	-0.062
	(0.003)	(0.004)	(0.008)	(0.006)	(0.006)	(0.014)
Pre-treatment avg.	0.982	0.982	0.982	1.397	1.397	1.397
R2	0.640	0.645	0.690	0.619	0.627	0.649
No. of obs.	776062	776062	776062	776062	776062	776062
Scholar FE	Y	Y	Y	Y	Y	Y
Year FE	Y	Y	Y	Y	Y	Y
Baseline Covariates*Year FE		Y			Y	
Entropy Balancing			Y			Y
**Panel C**	Total Publications			Total Citations		
Ties to China × Post	-0.012	-0.010	-0.014	-0.181	-0.107	-0.109
	(0.003)	(0.004)	(0.007)	(0.007)	(0.008)	(0.012)
Pre-treatment avg.	1.879	1.879	1.879	4.471	4.471	4.471
R2	0.757	0.757	0.797	0.685	0.687	0.727
No. of obs.	776062	776062	776062	776062	776062	776062
Scholar FE	Y	Y	Y	Y	Y	Y
Year FE	Y	Y	Y	Y	Y	Y
Baseline Covariates*Year FE		Y			Y	
Entropy Balancing			Y			Y

Note: All outcomes are log-transformed. For Columns (1) to (6), the models always control for scholar and year fixed effects. In Columns (2) and (5), we include the interactions between year dummies and four baseline covariates: 1) total number of publications in 2010-2014, 2) total citations in 2010-2014, 3) number of NIH-funded publications in 2010-2014, and 4) indicator for Asian researcher. In Columns (3) and (6), we use entropy balancing to balance all four covariates before running the regression. Standard errors are clustered at the scholar level.

3. More Heterogeneous Impacts

A. Effects by Pre-treatment Productivity. To investigate whether the treatment generates different impacts on scholars with varying levels of productivity, we divide the whole sample into two parts, based on whether a scholar's pre-treatment (2010–2014) total citation is above or below the median level (672 citations). In Table S14, odd columns report estimates using the group of highly productive scholars while even columns report estimates using the less productive group. The regression coefficients are smaller for the more productive group. However, scholars from this group have larger pre-treatment averages across all the outcomes. As explained in the main text, our outcomes are transformed by taking logarithm, and the regression estimates could be interpreted as percentages. Therefore, we can infer the impacts on the untransformed outcomes by multiplying the pre-treatment averages on the original scale with the effect estimates. Consider the total citations as an example. The pre-treatment averages are 5.152 and 2.784 for the two groups, respectively, which correspond to 171.777 and 15.184 citations on the original scale. Our estimates thus imply that the decline in citation would be $171.777 \times -0.034 = -5.840$ and $15.184 \times -0.104 = -1.579$ for the two groups. The highly productive group is more severely affected by the treatment in terms of the level of the outcome. This is true for the other outcomes as well.

Table S14. The Impacts on Productivity: Heterogeneity across Pre-treatment Productivity Levels

	(1)	(2)	(3)	(4)
**Panel A**	PubMed Publications		PubMed Citations	
Ties to China × Post	-0.013	-0.025	-0.026	-0.086
	(0.008)	(0.006)	(0.019)	(0.013)
Pre-treatment avg.	1.770	0.839	4.839	2.491
R2	0.769	0.576	0.676	0.511
No. of obs.	396543	396039	396543	396039
Scholar FE	Y	Y	Y	Y
Year FE	Y	Y	Y	Y
Entropy Balancing	Y	Y	Y	Y
Pre-treatment productivity	High	Low	High	Low
**Panel B**	Non-PubMed Publications		Non-PubMed Citations	
Ties to China × Post	0.014	-0.005	-0.038	-0.055
	(0.012)	(0.010)	(0.022)	(0.010)
Pre-treatment avg.	1.176	0.497	1.679	0.714
R2	0.682	0.511	0.659	0.486
No. of obs.	396543	396039	396543	396039
Scholar FE	Y	Y	Y	Y
Year FE	Y	Y	Y	Y
Entropy Balancing	Y	Y	Y	Y
Pre-treatment productivity	High	Low	High	Low
**Panel C**	Total Publications		Total Citations	
Ties to China × Post	-0.006	-0.023	-0.034	-0.104
	(0.009)	(0.010)	(0.018)	(0.013)
Pre-treatment avg.	2.186	1.118	5.152	2.784
R2	0.780	0.621	0.695	0.540
No. of obs.	396543	396039	396543	396039
Scholar FE	Y	Y	Y	Y
Year FE	Y	Y	Y	Y
Entropy Balancing	Y	Y	Y	Y
Pre-treatment productivity	High	Low	High	Low

Note: All outcomes are log-transformed. The models always control for scholar and year fixed effects, with all four covariates balanced by entropy balancing. Columns (1) and (3) present results for scholars whose pre-treatment productivity is above the median level, while Columns (2) and (4) present results for those whose pre-treatment productivity is below the median level. Standard errors are clustered at the scholar level.

B. Effects by Career Stage. We divide our sample into two parts to proxy career stages, based on whether a scholar's first publication was before or after 1996 (the median year for the first publication in our sample). In Table S15, odd and even columns report estimates using scholars below and above median. The estimates are similar in magnitude across the two groups.

Table S15. The Impacts on Productivity: Heterogeneity across Career Stages

	(1)	(2)	(3)	(4)
**Panel A**	PubMed Publications		PubMed Citations	
Ties to China × Post	-0.014	-0.014	-0.092	-0.081
	(0.006)	(0.008)	(0.013)	(0.020)
Pre-treatment avg.	1.386	1.589	3.927	4.339
R2	0.800	0.823	0.735	0.756
No. of obs.	395577	397005	395577	397005
Scholar FE	Y	Y	Y	Y
Year FE	Y	Y	Y	Y
Entropy Balancing	Y	Y	Y	Y
Career Stage	Early	Late	Early	Late
**Panel B**	Non-PubMed Publications		Non-PubMed Citations	
Ties to China × Post	0.032	0.006	-0.042	-0.057
	(0.006)	(0.015)	(0.012)	(0.024)
Pre-treatment avg.	0.883	1.054	1.304	1.474
R2	0.732	0.734	0.690	0.707
No. of obs.	395577	397005	395577	397005
Scholar FE	Y	Y	Y	Y
Year FE	Y	Y	Y	Y
Entropy Balancing	Y	Y	Y	Y
Career Stage	Early	Late	Early	Late
**Panel C**	Total Publications		Total Citations	
Ties to China × Post	0.004	-0.011	-0.095	-0.086
	(0.006)	(0.011)	(0.013)	(0.019)
Pre-treatment avg.	1.735	1.985	4.219	4.658
R2	0.821	0.829	0.755	0.772
No. of obs.	395577	397005	395577	397005
Scholar FE	Y	Y	Y	Y
Year FE	Y	Y	Y	Y
Entropy Balancing	Y	Y	Y	Y
Career Stage	Early	Late	Early	Late

Note: All outcomes are log-transformed. The models always control for scholar and year fixed effects, with all four covariates balanced by entropy balancing. Columns (1) and (3) present results for scholars who are at the early stage of their career, while Columns (2) and (4) present results for those who are at the late stage of their career. Standard errors are clustered at the scholar level.

4. More Results by Fields

A. Fields by Percentage of U.S.-CN Collaboration and NIH Funding.

Table S16. % U.S.-CN Collaboration and NIH Funding by Field

Top Fields by % U.S.-CN Collaboration		Top Fields by % NIH Funding	
Field Name	Percentage	Field Name	Percentage
Materials Eng.	18.2%	Biochemistry & Cell Biology	52.6%
Macromolecular & Materials Chemistry	14.5%	Medical Microbiology	48.7%
Nanotechnology	13.0%	Medical Physiology	46.9%
Physical Chemistry	13.0%	Bioinformatics & Computational Biology	44.7%
Earth Sci.	12.4%	Biological Psychology	43.3%
Engineering	11.9%	Neurosciences	43.1%
Condensed Matter Physics	11.2%	Medicinal & Biomolecular Chemistry	40.3%
Chemical Sci.	9.8%	Immunology	38.5%
Environmental Sci.	9.5%	Genetics	37.9%
Information & Computing Sci.	9.2%	Biological Sci.	37.0%
Inorganic Chemistry	8.9%	Microbiology	36.7%
Bioinformatics & Computational Biology	8.2%	Medical Biotechnology	36.6%
Medicinal & Biomolecular Chemistry	8.1%	Biomedical Eng.	36.5%
Medical Biotechnology	7.9%	Psychology	33.4%
Mathematical Sci.	7.6%	Epidemiology	33.3%
Genetics	7.5%	Pharmacology & Pharmaceutical Sci.	32.8%
Agricultural, Veterinary & Food Sci.	7.2%	Ophthalmology & Optometry	30.3%
Physical Sci.	6.9%	Public Health	30.2%
Organic Chemistry	6.7%	Clinical & Health Psychology	28.1%
Microbiology	6.6%	Paediatrics	26.9%
Biomedical Eng.	6.6%	Nutrition & Dietetics	26.1%
Biological Sci.	6.5%	Applied & Developmental Psychology	26.1%
Biochemistry & Cell Biology	6.4%	Organic Chemistry	24.9%
Ecology	5.4%	Social & Personality Psychology	24.2%
Medical Physiology	4.8%	Biomedical & Clinical Sci.	23.9%
Immunology	4.5%	Health Sci.	23.3%
Oncology & Carcinogenesis	4.4%	Health Services & Systems	21.0%
Epidemiology	4.3%	Reproductive Medicine	20.5%
Medical Microbiology	4.0%	Allied Health & Rehabilitation Science	18.6%
Neurosciences	3.8%	Cardiovascular Medicine & Haematology	18.1%
Ophthalmology & Optometry	3.8%	Clinical Sci.	17.9%
Pharmacology & Pharmaceutical Sci.	3.6%	Chemical Sci.	17.7%
Biological Psychology	3.4%	Sports Science & Exercise	15.8%
Dentistry	3.1%	Mathematical Sci.	15.5%
Biomedical & Clinical Sci.	3.1%	Human Society	15.3%
Nutrition& Dietetics	2.7%	Dentistry	14.8%
Psychology	2.5%	Inorganic Chemistry	14.7%
Public Health	2.5%	Nursing	13.6%
Clinical Sci.	2.2%	Information & Computing Sci.	13.3%
Reproductive Medicine	2.1%	Oncology & Carcinogenesis	12.8%
Cardiovascular Medicine & Haematology	2.0%	Engineering	12.2%
Health Sci.	2.0%	Physical Chemistry	8.1%
Social & Personality Psychology	1.9%	Macromolecular & Materials Chemistry	8.1%
Human Society	1.9%	Environmental Sci.	8.1%
Applied & Developmental Psychology	1.6%	Nanotechnology	7.5%
Allied Health & Rehabilitation Science	1.4%	Agricultural, Veterinary & Food Sci.	6.4%
Health Services & Systems	1.4%	Ecology	6.2%
Clinical & Health Psychology	1.4%	Physical Sci.	4.8%
Sports Science & Exercise	1.3%	Materials Eng.	3.2%
Paediatrics	1.2%	Earth Sci.	2.2%
Nursing	1.0%	Condensed Matter Physics	1.6%

References

1. J Mervis, Pall of suspicion. *Science* (2023).
2. K Imai, K Khanna, Improving ecological inference by predicting individual ethnicity from voter registration records. *Polit. Analysis* **24**, 263–272 (2016).
3. Y Xie, X Lin, J Li, Q He, J Huang, Caught in the crossfire: Fears of Chinese–American scientists. *Proc. Natl. Acad. Sci.* **120**, e2216248120 (2023).
